# Dorsal CA1 lesions of the hippocampus impact mating tactics in prairie voles by shifting non-monogamous males’ use of space to resemble monogamous males

**DOI:** 10.3389/fnbeh.2024.1355807

**Published:** 2024-02-26

**Authors:** Lindsay L. Sailer, Caitlyn J. Finton, Pooja P. Patel, Steven M. Bogdanowicz, Alexander G. Ophir

**Affiliations:** ^1^Department of Psychology, Cornell University, Ithaca, NY, United States; ^2^Department of Ecology and Evolutionary Biology, Cornell University, Ithaca, NY, United States

**Keywords:** dCA1, mating tactics, prairie vole, reproductive success, space use, spatial cognition

## Abstract

Alternative mating tactics within mating systems are characterized by discrete patterns of spatio-temporal overlap with same-and opposite-sex conspecifics and mating-relevant outcomes. Socially monogamous “residents” maintain relatively small home range sizes, have territories that almost exclusively overlap with their mating partners, and are more likely to produce offspring than non-bonded “wandering” conspecifics. Because mating tactics appear to be so closely tied to patterns of space use, differences in spatial cognitive abilities might differentially impact individual males’ decisions to adopt a particular mating tactic and/or how efficient they are within their chosen mating tactic. Yet few studies have considered how the hippocampus, a brain region important for encoding cognitive maps and for processing contextual information, might impact how individuals adopt mating tactics or the spatio-temporal behaviors closely associated with them. We assessed the impact of lesions to the dorsal CA1 (dCA1) region of the hippocampus on male prairie vole space use, reproductive success, and mating tactics in semi-natural outdoor field conditions. Interestingly, dCA1 lesions did not impact the proportion of males that adopted resident or wandering mating tactics, and dCA1 lesions did not impact a male’s ability to form a pair bond in the lab. In contrast, we found that lesioning the dCA1 shifted the home range size of reproductively successful and unsuccessful males. Furthermore, we found that patterns of space use among residents were unaffected by dCA1 lesions, whereas wanderers with dCA1 lesions showed pronounced reductions of their space use habits and resembled non-lesioned residents. Collectively, our study supports the hypothesis that wanderer male prairie voles rely on dCA1-mediated spatial cognition to navigate their world in a way that resident males do not. Such differences might have implications for how individuals efficiently attract and defend mates, obtain resources, defend territories, and outcompete rivals.

## Introduction

Mating systems (i.e., monogamy, polygamy, etc.) describe the most common reproductive strategy observed within a population of animals (Shuster and Wade, 2003). They are often defined by the number of mates males and females have and are, therefore, an emergent property of the individual reproductive choices observed within populations of a given species. Individual reproductive tactics that comprise mating systems are based on each individual’s assessment of the ecological and social landscape (Oliveira et al., 2008). Thus, individual reproductive decisions to adopt a particular mating tactic are based on numerous forms of information and information processing, including spatial cognition.

Spatial cognition serves a critical role in determining how animals accumulate opportunities to reproduce. Indeed, spatial cognition enables animals to learn the identity and distribution of conspecifics in space and then use this information to assess and navigate their social landscape, which can affect reproductive success (Ophir, 2017; Elson et al., 2024). For instance, spatial cognition has the potential to impact reproductive decisions by benefiting animals that must cover large areas of space to locate multiple mates, thereby allowing polygamous males to remember and effectively guard the location of those potential mates (Gaulin, 1992). Alternatively, males might also rely on spatial cognition to maximize their reproductive success within a monogamous mating system by enhancing the ability to track and defend territorial boundaries and the conspecifics that reside in and around that territory (Phelps and Ophir, 2009; Ophir, 2017). It should be noted that these two possible ways in which spatial cognition can impact reproductive decision-making are not mutually exclusive. Taken together, identifying the social and cognitive factors that shape individual mating decisions is necessary to predict reproductive success and to understand how individual decisions contribute to and shape social organization.

Field and laboratory studies have supported the link between spatial cognition and mating system within and between a variety of species (Gaulin and Fitzgerald, 1986; Sherry et al., 1992; Clint et al., 2012; Jasarevic et al., 2012). For example, males of the polygynous deer mouse (*Peromyscus maniculatus*) outperform males of the monogamous California mouse (*P. californicus insignis*) in a spatial memory test (Jasarevic et al., 2012), potentially indicating a greater reliance on spatial cognition among males of polygynous species. Although the benefits of tracking conspecifics, and potential mates in particular, are presumably very high for polygamous males (Gaulin, 1992), there is also a clear autocorrelation with home range size and use of that larger space. Thus, the difference in spatial cognitive performance could reflect experience (Maguire et al., 2000) rather than adaptive specialization for spatial cognition between species based on the reproductive strategies that underlie mating systems. Moreover, to assume that monogamous males rely on spatial cognition any less than polygamous males ignores the fact that monogamous males typically rely heavily on spatial cognition to define and defend home territories, track and mate guard their partners, and track the movements of conspecifics (either other male competitors or additional extra-pair mates). Indeed, some evidence has supported the idea that monogamous males should also heavily rely on spatial cognition, even if it is used in ways that differ from polygamous males (Phelps and Ophir, 2009; Ophir, 2017; Rice et al., 2022).

The hippocampus (HPC) is well known for its involvement in spatial cognition (Zola-Morgan and Squire, 1993; Rolls, 1996; Strange et al., 2014). The HPC is comprised of the dorsal and ventral regions that are, by and large, functionally distinct (Strange et al., 2014). The dorsal HPC is commonly associated with subserving episodic memory, spatial maps, and navigation, whereas the ventral HPC appears to be particularly important for emotional memory, affect, and stress (Moser and Moser, 1998; Fanselow and Dong, 2010). Indeed, spatial cognition is affected by dorsal HPC lesions, but not ventral HPC lesions (Moser et al., 1993). Within the dorsal and ventral regions, different sub-structures seem to also have distinct roles. The CA1 region is essential for spatial learning and memory (Tsien et al., 1996), whereas the CA2 region of the HPC appears to play an important role in social identity learning and memory (Hitti and Siegelbaum, 2014; Tzakis and Holahan, 2019). However, there appears to be variation in function along the dorsal-ventral axis, such that the dorsal CA1 processes spatial memory, whereas the ventral CA1 is an important site for social memory processing (Okuyama et al., 2016).

Although they are rarely thought of in such terms, mating systems are inherently spatially cognitive challenges for animals (Ophir, 2017). Indeed, many common metrics used to define mating systems, and mating tactics therein, are effectively measures of how animals use space. Furthermore, differences in HPC neuroanatomy of species with different mating systems could reflect variation within and between species in terms of how they use space. For example, males of polygamous species typically have larger home ranges and territories compared to females, presumably reflecting the strategy to locate and/or monopolize as many females as they can (Emlen and Oring, 1977; Shuster and Wade, 2003). This use of larger territories might reflect a significant demand on spatial cognition (Jacobs et al., 1990; Sherry et al., 1992; Jacobs and Spencer, 1994; Yaskin, 2013). Supporting this hypothesis, male Merriam’s kangaroo rats (*Dipodomys merriami*) and bannertail kangaroo rats (*D. spectabilis*) have a larger HPC volume than females (Jacobs and Spencer, 1994). Similarly, male meadow voles (*Microtus pennsylannicus*) have larger HPC volumes and home ranges than females (Jacobs et al., 1990). In contrast, monogamous species usually do not show this sexual dimorphism in HPC volume or home range size. For instance, HPC volume and home range size do not differ among male and female pine voles (*M. pinetorum*) (Jacobs et al., 1990) or male and female prairie voles (*M. ochrogaster*) (Ophir et al., 2008b; Rice et al., 2017). Notably, however, male prairie voles outperform females in the Morris water maze task, and males demonstrate a lower density of oxytocin receptors within the CA1 region of the dorsal HPC (Rice et al., 2017), which might enhance HPC function in this HPC dependent task (Mcewen, 2004). The associations between HPC neuroanatomy and function, and variation in space use and the mating tactics associated with space use suggests that spatial cognition might shape the reproductive decisions that individuals within a population make (Ophir, 2017).

Prairie voles are socially monogamous rodents that readily form long-lasting pair bonds with a mating partner (Gavish et al., 1981; Getz et al., 1981; Carter and Getz, 1993). In the field, prairie voles tend to live in pairs and the majority of male prairie voles (~60–75%) adopt a socially monogamous mating tactic (Getz et al., 1993; McGuire et al., 2013; Madrid et al., 2020). Males adopting this so-called ‘resident’ mating tactic have home ranges that closely overlap with the home range of just one female (i.e., their partner) and are believed to defend their home range from intruding male competitors (Getz and McGuire, 1993; Solomon and Jacquot, 2002; Ophir et al., 2008c). Moreover, the typical resident pattern of space use includes relatively small (defendable) home ranges that show minimal overlap with conspecifics other than their partner, but a high degree of spatial overlap between their own home range and that of their partner. Notably, variation within resident behavior exists. Resident males may mate exclusively with their partner (i.e., true residents) or seek extra-pair copulations outside of their pair bond (i.e., roving residents) (Rice et al., 2017; Madrid et al., 2020). The degree to which males rove or not appears to be contextual, depending on the social composition of the population and the proportion of other rovers within that population (Rice et al., 2018). The decision to rove effectively or not might therefore also be influenced by an individual’s ability to navigate space and/or track conspecifics within space.

In contrast to residents, some males (~25–30%) exhibit a “wandering” mating tactic. Wandering males typically occupy a much larger home range than residents, which overlap with the home ranges of multiple males and females (Getz and McGuire, 1993; Solomon and Jacquot, 2002; Ophir et al., 2008b; McGuire et al., 2013). Additionally, wanderers do not appear to defend their home range and presumably do not form pair bonds (Getz and McGuire, 1993; Solomon and Jacquot, 2002; Ophir et al., 2008c; McGuire et al., 2013). In other words, although wanderer males appear to use space more like males within a polygynous mating system (i.e., large home ranges that overlap many females), they do not appear to be territorial in their use of space (i.e., exclude males or manage defendable-sized territories). Thus, the selective pressures that might enhance spatial cognition could favor wanderers, roving residents, true residents, or all three but for different reasons.

Considering the degree to which establishing and defending a home range or tracking conspecifics in space is contextual and fundamentally based on navigation, and considering the link between these forms of cognition and HPC function, we tested the hypothesis that male prairie vole space use, reproductive success, and mating tactics are influenced by the HPC. Specifically, we sought to determine if lesioning the HPC would influence the chosen mating tactics (residents and wanderers) that males would adopt while living in a semi-naturalistic outdoor field enclosure. We specifically lesioned the dorsal CA1 subregion (dCA1) within the HPC, because the dCA1 is known to be particularly important for spatial navigation and contextual learning and memory (Moser et al., 1993; Tsien et al., 1996; Fanselow and Dong, 2010). We predicted that if HPC function promotes residency behavior (i.e., enhances territorial behaviors), then lesions of the dCA1 would reduce the probability of adopting residency-like behavior, increase home range size, increase home range overlaps, and could impact reproductive success. Therefore, if HPC function promotes residency behavior, dCA1 lesions would produce more animals that resemble the typical ‘wanderer’ phenotype. On the other hand, if HPC function promotes wandering behavior (i.e., enhances spatial navigation of large areas and enhances navigation of the socio-spatial landscape), then lesions of the dCA1 would increase the probability of adopting residency-like behavior, decrease home range size, decrease home range overlaps, and could impact reproductive success. In other words, if HPC function promotes wandering behavior, dCA1 lesions would produce more animals that resemble the typical ‘resident’ phenotype.

## Methods

### Animals

All animals (110 males and 115 females, see below) in this study were the laboratory-bred offspring from unrelated pairs of F1 or wild-caught prairie voles in our breeding colony. These wild-caught breeders were originally trapped in Urbana-Champaign, IL, USA. We weaned pups at 21 days old and housed them with same-sex siblings in standard polycarbonate cages (46.5 × 25 × 15.5 cm) lined with Sani-chip bedding and containing nesting material. We kept animals on a 14:10 light:dark cycle and provided rodent chow (Laboratory Rodent Diet 5,001, LabDiet, St. Louis, MO, USA) and water *ad libitum*. Ambient temperature was maintained at 20°C (±2°C). All animals were sexually naïve, between 60 and 80 days old (i.e., adulthood), and individually marked with a small metal self-piercing and self-locking ear-tag that was laser etched with a unique four-digit number for individual identification (S. Roestenburg, Riverton, UT, U.S.A.). Animals used in this experiment were unrelated and unfamiliar with one another unless otherwise noted. Sex was determined based on external genitalia. All procedures were approved by and in compliance with the Institutional Animal Care and Use Committee of Cornell University (Protocol 2013–0102).

### Hippocampal lesion surgery

We assigned 90 unrelated males to either sham (*n* = 45) or hippocampal lesion (*n* = 45) conditions. Male subjects were anaesthetized with 4% isoflurane mixed with pure oxygen (1 L/min) initially and after 2 min anaesthetized with a 2% isoflurane mix throughout the remainder of the surgery. Subjects were then secured into a stereotaxic apparatus (Kopf Instruments). The scalp was shaved and scrubbed with povidone-iodine (Purdue Products), and ophthalmic ointment (Henry Schein) was applied to prevent drying of the eyes during surgery. Subjects received four injections (two injections per side) of either ibotenic acid (lesion condition; 0.1 uL/injection, 10 ug/mL of freshly made ibotenic acid in 0.9% saline) or vehicle (sham condition; 0.9% saline) in their dCA1. After the injection of the full volume, the needle was left in place for 5 min and was then retracted slowly at approximately 1 mm/min to prevent any liquid backflow. Access holes were drilled bilaterally into the skull, and a glass pipette connected to a 1.0 μL syringe (Hamilton Laboratory Products, Reno, NY) with tubing was lowered to the verified coordinates (from bregma: *site 1*: AP –1.2 mm, ML 1.4 mm, DV 1.8 mm; *site 2*: AP –2.2 mm, ML 2.5 mm, DV 1.8 mm). Once injections were complete, the incision was swabbed with povidone-iodine again, sutured, and subjects were allowed to recover on a heated pad until normal locomotor behavior resumed. After surgery, subjects were single-housed in polycarbonate cages (29 × 18 × 13 cm) and given 300 mg/kg/day liquid acetaminophen in water for 2 days and monitored daily. All animals recovered without incident. Experiments began 2 weeks post-surgery.

### Semi-natural fieldwork

Ninety males (*n* = 45 sham, *n* = 45 lesion, detailed above) and 90 females were used in the field work component of this experiment. All animals lived freely in the outdoor enclosures described below, where food and water were naturally available. We conducted a total of nine replicates between early June and mid-August in 2018 (*n* = 4) and 2019 (*n* = 5). Each field enclosure replicate contained 10 females, 5 lesion males, and 5 sham males. In 2018, replicates occurred in sequence in the same enclosure for June and July; two replicates were run in tandem in separate fields during August. In 2019, two replicates were run simultaneously during June and July; followed by a single replicate in August.

**Figure 1:**
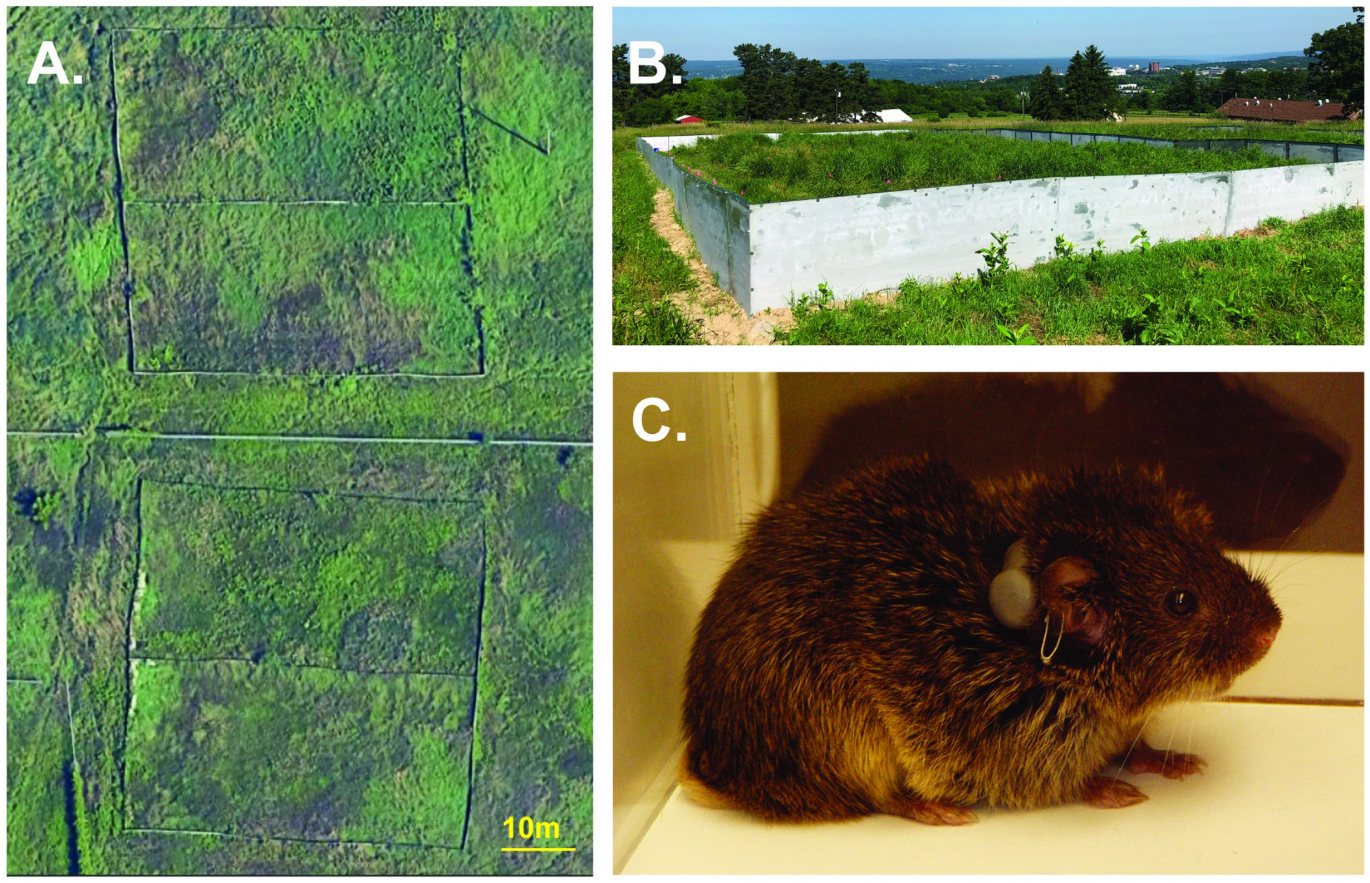
Semi-natural outdoor enclosures. **(A)** Overhead satellite view of the field enclosures. **(B)** Ground view of one field enclosure. **(C)** Male prairie vole with radio-collar and ear tag.

The field enclosures were located in Ithaca, New York, U.S.A., each with an identical construction design and habitat. The enclosure size was designed to ensure the population density in our experiment, 200 voles/ha, would be within the parameters of natural population densities for prairie voles in the wild (11–624 voles/ha; Getz et al., 1993). Enclosures (approximately 40 × 20 m; Figures 1A,B) were constructed of aluminum walls and powder-coated steel tube frames. The walls extended ~75 cm above and below ground to prevent subjects escaping the enclosures and to prevent other terrestrial animals from entering the enclosures. All of the enclosures contained the same soil and similar distribution and composition of vegetation including dicots and mixed pasture grasses (i.e., fescue, brome, and rye) that are consistent with the prairie vole’s natural habitat. Survey flags (10.2 × 12.7 cm, with a 53 cm wire pole) were placed in each enclosure in a 33 m × 18 m grid with 3-m spacing between flags.

For each replicate, we radio-collared each vole with a 1.9 g transmitter (BD-2C, Holohil Systems Ltd., Carp, Ontario, Canada) affixed with a weather resistant zip-tie collar (112 × 2.5 mm) (Figure 1C) to track movements and assess their individual patterns of space use (Ophir et al., 2007, 2008a,c, 2012; Zheng et al., 2013; Rice et al., 2022). Animals were closely monitored after collaring to ensure they did not show signs of discomfort. Collars were removed from animals that showed discomfort and replaced the next day. Animals were transported from the laboratory breeding colony and released into field enclosures 2 days after being radio-collared. We first released all females together in 1 day, then we released all males the following day. Animals were released at different points along the same edge of the enclosure, with 3 meters between each release point.

### Radiotracking

We determined the location (hereafter, a ‘fix’) of each animal twice a day for 18 days. To avoid potential temporally-dependent habits in space use, we varied the time at which we recorded locational fixes across the day during daylight hours (between 07:00 and 18:00 h EDT). One fix was always collected in the morning (before 12:00 h); the other was collected in the afternoon (after 13:00 h) at least 1 h after the morning fix. We tracked animals with a Yagi radio-telemetry antenna (3 Ele Fldg, Advanced Telemetry Systems, Isanti, MN) and receiver (R4000, Advanced Telemetry Systems, Isanti, MN, USA). We used the survey flag grid to record each vole location.

### Measuring space use

We used RANGES 9 software (Anatrack Ltd., Wareham, Dorset, UK) to assess how individuals used space in the semi-natural field enclosures. We converted each animal’s daily locations on the field grid plot to *X* and *Y* coordinates, and then characterized each animal’s home range with minimum convex polygons (MCPs). MCPs are the simplest and most common measure of space use and have several advantages over other space use assessments like kernel estimators (White and Garrott, 1990; Powell, 2000; Row and Blouin-Demers, 2006).

In some instances, fixes represent outlier excursions outside the ‘core’ home range. Therefore, we determined the appropriate threshold level of fix exclusion to ensure we were describing the core home range appropriately. To this end, we fit a curve to the average peeled polygon home ranges, which removes the locations furthest from the harmonic mean centers of the home ranges at 5% intervals. We began at 100% fix inclusion and decreased at 5% intervals to 25% fix inclusion. We visually assessed the inflection point when the outermost fixes stopped biasing the MCP estimate of home ranges, which fell between 70 and 80% cores. We therefore chose 75% fix inclusion to describe an individual’s primary home range. Notably, this 75% threshold for fix exclusion is consistent with other studies that have used the same methods to determine the core home range for each individual (Ophir et al., 2008b, 2012; Rice et al., 2022), indicating this is a reliable and robust threshold. Indeed, MCPs with 75% cores eliminates outliers and captures the space a prairie vole is most likely to occupy (White and Garrott, 1990).

We used these home ranges to determine the amount of home range overlap each male had with females and other males. The extent to which a male’s home range overlapped with one female more than all other females was the basis for our space use determination (Ophir et al., 2008a; see below). We also calculated the overall area of each home range and the number of individuals (same-sex, opposite-sex, or total) that overlapped a given individual’s home range.

### Determining mating tactic

We classified males as paired residents or non-paired wanderers based on home range MCPs. This determination was based on the idea that a member of a pair should mutually share space with one animal of the opposite sex more than any other of that sex, and vice versa. This approach enabled us to quantify commonly understood qualitative definitions of paired animals (Ostfeld, 1990; Rice et al., 2022).

We first used RANGES 9 to calculate the ‘Pairwise Encounter Rate’ (PER) for each animal. These values estimate the likelihood of individual interactions between a subject and every other animal within an enclosure (Ophir et al., 2008a). We calculated the PER for each possible pair by taking the product of the proportion of home range overlap between every male and female.


$$\mathrm{PER}=\frac{\left(\%\mathrm{overlap}\;{\mathrm{male}}_{i}|{\mathrm{female}}_{j}\right)\;x\;\left(\%\mathrm{overlap}\;{\mathrm{female}}_{j}|{\mathrm{male}}_{i}\right)}{100}$$

We then divided this PER by the sum of all possible PERs with opposite-sex individuals to calculate an individual’s ‘Relative Encounter Rate’ (RER) with opposite sex individuals.


$$For\;\mathrm{each}\;\mathrm{individual}\left(i\right),{RER}_{i}=\frac{{\mathrm{PER}}_{i}}{\sum_{k=1}^{n}{\mathrm{PER}}_{k}}$$

An individual’s RER estimates the probability of two individuals encountering each other based on the proportion of home range they share, given all other individuals in an enclosure.

We calculated RERs for all possible male and female pairs. A minimum RER score of 0.0 indicated that the female occupied none of a particular male’s opposite-sex overlap area and they were unlikely to interact. A maximum RER score of 1.0 indicated that a female comprised all of a particular male’s opposite-sex overlap area and they were highly likely to interact.

We determined mating tactic (paired residents or unpaired wanderers) by comparing all possible RER combinations between all males and females within an enclosure. Pair-bonded voles share and mutually defend a home range (Getz and McGuire, 1993) and pairs of animals should encounter each other more than other individuals in a population. Therefore, we considered a male and a female to be paired if they both had a RER equal to or greater than 0.5 for each other. Any male that did not meet this criterion was considered to not be paired with a female. The largest RER for a given male (i.e., RER_PRIMARY_) was used as a continuous measure of social fidelity. Specifically, RER_PRIMARY_ was defined as the RER between a male and the female with whom the male had the most extensive home range overlap. Similarly, we defined the RER_SECONDARY_ to be the RER between a male and the female with whom the male had the second-most extensive home range overlap. Residents are expected to have very a large RER_PRIMARY_, and a substantially smaller RER_SECONDARY_. In contrast, wanderers are expected to have similarly sized RER_PRIMARY_ and RER_SECONDARY_, where the RER_PRIMARY_ should be smaller than those of residents and RER_SECONDARY_ should be larger than those of residents. Thus, these measures enable the comparison of overall patterns of space use between resident and wanderer tactics.

### Tissue collection

Animals were live-trapped out of the enclosures after 18 days using a mixture of Fitch traps and Sherman traps baited with sunflower seeds and oats. Animals were briefly transported in standard polycarbonate cages (29 cm × 18 cm × 13 cm) to our field laboratory (located approximately 125 m from the enclosures) and temporarily housed for up to 3 h. We then humanely euthanized the animals with CO2 inhalation. Brains from the males were immediately removed and flash-frozen using powdered dry ice, and then stored at −80°C until cryosectioning for lesion quantification. We also collected tissues from all males, females, and embryos. For all males and females, tail clippings were collected prior to release into the enclosures. We also collected leg muscle tissue from each adult animal retrieved from the field enclosure. Fetuses were extracted from the mothers’ uterine horns and placed individually on a clean (DNA-free) surface. Next, we removed the embryonic sac and placenta, and measured the crown–rump length to estimate embryo age (Ophir et al., 2013). All tissue (tail clips, leg muscle, and fetuses) was stored in 70% ethanol and frozen at −80°C. DNA was extracted from tail clips, muscle tissue, and fetuses following standard Maxwell Tissue DNA Purification Kit protocols (AS1030, Promega Madison, WI) for parentage analysis. When possible, DNA from muscle tissue was used preferentially, however tail tissue was used when an animal was not recovered (see below).

### Lesion quantification

**Figure 2:**
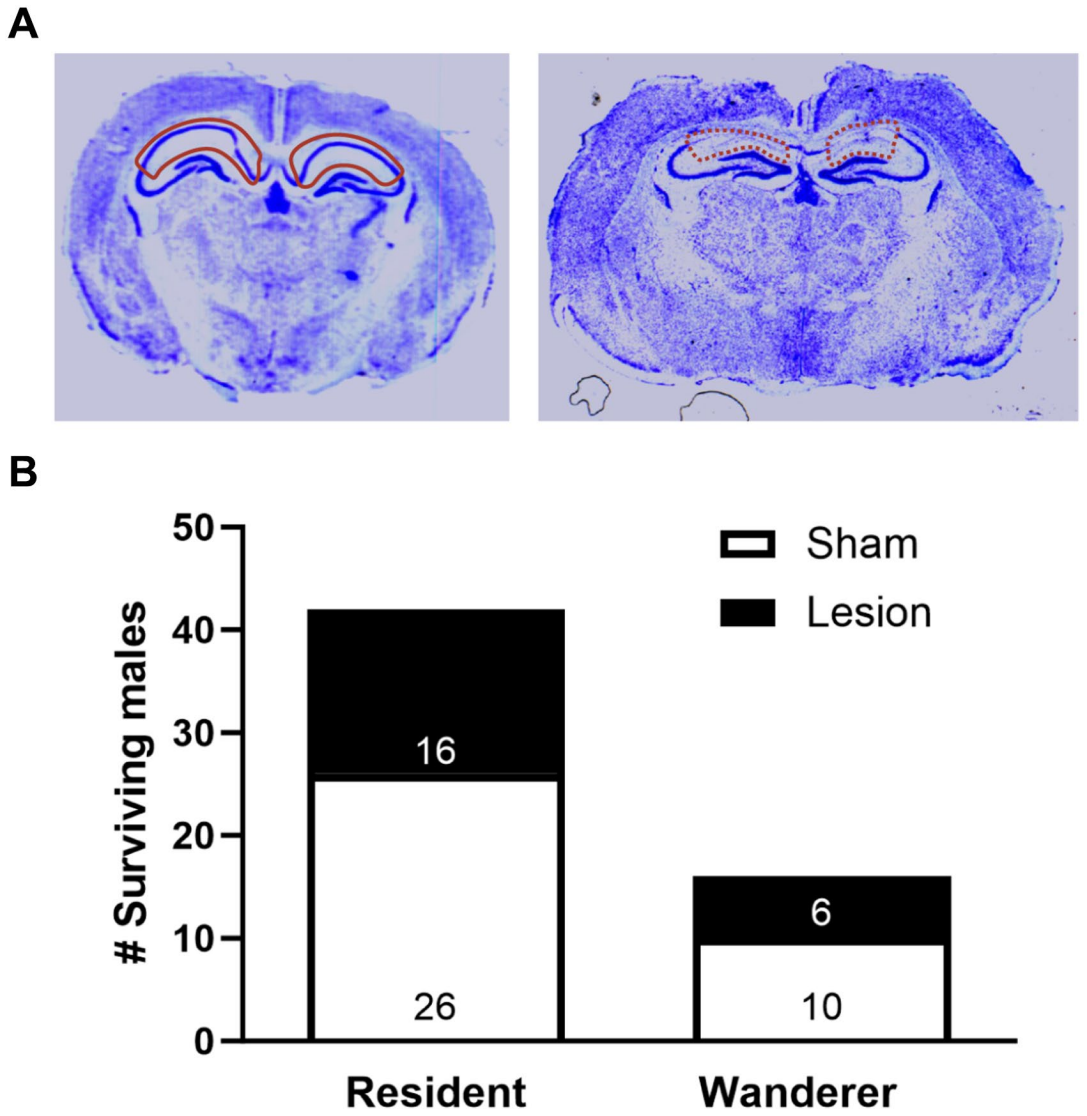
Impact of dCA1 lesions on prairie vole mating tactics. **(A)** Representative cresyl violet-stained coronal brain sections of dCA1 sham (left, solid red outline) and lesioned (right, dashed red outline) subjects. The image here portrays an animal with a dCA1 lesion of 53.4%. **(B)** Number of recovered resident and wanderer male prairie voles that either received dCA1 sham (white) or dCA1 lesions (black). Data are pooled across field enclosures.

Brains were cryosectioned (20 um thick) at −20°C in a cryostat. We collected sections beginning at the rostral HPC (AP -0.88 mm to −3.38 mm from Bregma) and immediately mounted the sections on slides (Figure 2A). In brief, brains were thawed and air-dried overnight, bathed in a series of 100% EtOH, 95% EtOH, 75% EtOH for 2 min. The slides were then bathed in 0.5% cresyl violet with 1 M acetyl acetate for 90 min and then re-hydrated in a reverse series of EtOH baths (75, 95, 100%) for 2 min. Finally, slides were washed in Citrisolv (Fisher Scientific) for 2 min, and air-dried. The slides were then prepared with permount (Electron Microscopy Sciences, Hatfield, PA), coverslipped, and allowed to cure for 2 days.

To determine the percentage of dCA1 lesion for each animal, we analyzed the slides using ImageJ (National Institutes of Health, Bethesda, Maryland, USA) and identified the dCA1 using reliable landmarks associated with this area (e.g., corpus callosum, anterior commissure, etc.). To calculate total dCA1 size, we outlined the dCA1 using the select tool, and used the ‘measure’ function to calculate area. We then repeated this process, outlining only the lesioned region on the same section. This was repeated along the rostral-caudal axis as described previously (Rice et al., 2017). Every section containing the dCA1 was analyzed (~17 slices/brain). We then calculated a percentage of the dCA1 that was lesioned for each animal by taking the sum of the area of the dCA1 across all sections, and the sum of the area of the lesion on each section. We then divided the total area of lesion by the total area of the dCA1.

A minimal lesion volume of 20% to the dorsal hippocampus can impair spatial memory (Moser et al., 1993). Therefore, we only considered subjects that had at least 25% of their dCA1 lesioned in our study. None of our dependent variables significantly correlated with percent lesion, with the exception of home range size (*R*^2^ = 0.43, *p* = 0.05), which made us confident that ≥25% was an appropriate threshold.

### Parentage analysis

#### Development of microsatellite loci for multiplex PCR

We developed 41 polymorphic microsatellite loci for multiplex PCR from a genomic DNA library enriched for tetrameric repeats (D'Aloia et al., 2017; Rueger et al., 2021). Loci were amplified (see Table 1 for locus-specific primer sequences) for all males, females, and fetuses using the QIAGEN Multiplex PCR Kit. We pooled samples across multiplexes and used Illumina’s S5 and N7 Nextera primers to barcode each individual. All barcoded individuals were then pooled to prepare a sequencing library, which was then size-selected with 0.7X Ampure XP (Beckman Coulter). The library was sequenced on a MiSeq (Illumina) in paired-end 250-bp mode at Cornell University’s BioResource Center as described previously (D'Aloia et al., 2017; Rueger et al., 2021).

**Table 1:** Polymorphic microsatellite markers developed for *Microtus ochrogaster*.

Locus	Forward	Reverse
Moch_c1_3	AGCTGGTATAATCACAGGCCTGC	TTGGCTATGCACATGTACTACCAG
Moch_c1_4	TCACCATCCACCATCATTTACTGG	GGCAATAATCAGAGTGTTGCTGGG
Moch_c1_5	TTTGCCTGTTACAACCATGCCTG	TCCATTAACTTGTCTCCGTCTTGAC
Moch_c1_6	CAACTCCCAGTCCTTGATCCAGG	GCGACAGATTGAGAACCTGCATC
Moch_c2_1	TGCCCTTCTGACTTCTGTTCACC	ATGAGATCTGGTGCCCTCTTCTG
Moch_c2_3	ACCTCTCTGTCAAATACCACATGTC	AGATCTGGTGCCCTCTTCTGG
Moch_c2_4	ACCGGAAGTTTGAAGTCCTTGTG	TGGTGCCCAAATGCTATATGGAG
Moch_c2_5	CCCAGGTTTAGGCAGACATAGGG	GGTTCCAAAGCTACAGAGAAACCC
Moch_c4_1	TCGAATTCAGGTCACCAGGCTTG	TGATTGCTCTTAGGGCTGACGAG
Moch_c4_2	GCCTGCCATGAAGAGCCCTATC	AAGAGTCAAGAACAGGCTGGAGG
Moch_c4_3	ACCGCTAGTGAAAGATGGCCAG	GAGTGAGTTCCAGGACAGGCTC
Moch_c4_4	ATTCCCTCTCCCGTCCACATTC	AGAGTCCCACAAGAACACCAAAC
Moch_c5_1	CCCTCGCCACGGTTAGCAAG	TGATTGCTCTTTGGGCTGACGAG
Moch_c5_3	ACACCTGAAAGTACCTCTCCTGC	CAGGTGAGCAATGATGAGATGGG
Moch_c5_4	GTGTAGGAAAGAGGGAGGTGACG	AGCAAACGTGTCAATCTCATACCC
Moch_c5_5	AGACCCTACCTTACCCTCCTCAC	AGACTCCTGCAGATTGGCCTC
Moch_c6_1	ACAGGTTCCACAGAGAAACCCAG	TGAGACAGGATTTCTCACAGGCC
Moch_c6_3	ACTGTGTGCATTCAAGATCTCTCC	GGAGAGTCAAGGGCTACACAAATAG
Moch_c6_5	GCACTTGGGAGGCAGACG	AGGTCAGTGCTTTGTATGGCTTG
Moch_c7_1	TGATCCAATGACCTCTTCTGGCC	ATGTGGTGTTCTAAGGACCTGCC
Moch_c7_2	ATGCTTCTCTGTGAGGTACGAAC	CCCTACCTAAGCCAGTTGAGGTG
Moch_c7_3	TCTCTGCGTCGCCTCATTAACTC	CTCCCTCCTAGACCTGCCCTATG
Moch_c7_4	CACCAGGAACACACGTGATACAC	CGCCCGGCTTATTTGTTCCATTC
Moch_c7_5	CACCACACACACTGACTGTCTG	CAGGTCTCTCACGGAAACTGGAG
Moch_c8_1	ACCTTGAACTCCTGATGTTCCCG	TAGGAGGGTCTCTGTGAGTCTGG
Moch_c8_2	TAGTATGCTTGTGGACACGTGTG	GGAGCCAGAGACAGGTGAATCTC
Moch_c8_3	TCAGGAAGAGCAAGCAGTCAGTG	GAACTCCAAGTCACCCTCTTCTG
Moch_c8_5	AGCCATGATCCATTTCTTGACCC	GCACGCTCACACACGAATAAATG
Moch_c10_1	CACGAATGCAGCCCAATTGATTTC	CAAGAGCTAGTTCCAGGACAGGC
Moch_c10_2	GGAAAGAGAACGGAAGGATGGAC	CGTTATGACCACTACTGATCCCTC
Moch_c10_3	AGTGGGCTGGTTGACTGAGTTAG	AAACTCAAGCGACCAACCTGTC
Moch_c10_4	GGTGGTGTGCATCTTTAACAGGG	GTTGTATGATAAGGCCTCTCTTGGG
Moch_c10_5	CCAGTCTCAGGAGTCTTCGGATG	GCATTTCCTGTCCCTGTGGTGG
Moch_c15_1	CCTGCCGATGTGTGTTTAACCTG	TCTCTGTGAGTTTGAGGCCATCC
Moch_c15_2	TCCAGGGAAGCATGAGACCATTC	AGACTAGTGATTTGCCTGCCTCG
Moch_c15_3	GTCTGAGCATGGAGAAGCAATGG	GTCACACCCATGATCCCTGGAAC
Moch_c16_1	ACTCTTCATCTGCTAGACCAAGGAC	CCGCACATTGGGAAGTGTCATTC
Moch_c16_2	CAGTATTTGTCTCCTTCGGCAGC	GGGTGTCCTGTATATGTGCTTCG
Moch_c16_3	AGATAGCCTCAGATCAATGGCTTG	CCAGACACACACACATGGTTTACG
Moch_c16_4	GCTGATGACCTGAATGACCCAAG	GCCTTTAATCCCAGTGCTCGGG
Moch_c17_2	CCGCAGAGTCTCAAGCATTTCAC	AGGTGTATCTGTGAGGACCCATAC

Forward and reverse primer sequences (5’à’3′).

#### Data processing

We used a python script (amplicon.py^1^) to extract reads form the MiSeq run and assign genotypes at each microsatellite locus and individual. Default commands were used except the following: -c 1, −a 0.005, −l 150. We also explored two-reads ratios (−r command) for calling heterozygotes: the default of –r 20 as well as –r 40. A minimum of two reads were required for each allele; otherwise, the diploid genotype was recoded as missing data. To retain only the highest quality markers and individuals, we first excluded loci missing >20% of individuals, then individuals with >20% missing loci. After the filters were applied, 428 individuals remained, which were analyzed at 41 loci (Table 1) in Hardy–Weinberg equilibrium.

#### Parentage assignment

We assigned maternity and paternity to fetuses using CERVUS 2.0 (Marshall et al., 1998) for each enclosure. Because there were only 10 females and 10 males per enclosure replicate, no more than 10 candidate mothers and 10 candidate fathers were possible. Mothers were known with 100% confidence because embryos were directly extracted from them. At least 20 typed loci were required per individual to assign parentage, the error rate was assumed to be 0.001%, confidence intervals were placed at 80% and 95%, and simulations were run for 10,000 cycles. We accepted maternity and paternity assignments only if the delta values (log_e_ likelihood ratio of most likely to second-most likely parent) were equal to or greater than 0.69, corresponding to the value at which the most likely parent was at least twice as likely at the second-most likely parent. Omitting this latter criterion would have caused some equivocal parentage assignments with inflated confidence, and would tend to overestimate the abundance of extra-pair fertilizations and multiple paternity. By combining these data with our space use information, we were able to estimate the number and nature of successful mating.

#### Data analysis

Data were analyzed using R Statistical Software (v4.0.3; R Core Team, 2016) using linear mixed model (LMM) frameworks with the packages lme4 (Bates et al., 2015) and lmerTest (Kuznetsova et al., 2020). Significant main effects and interactions were followed by pairwise *post hoc* multiple comparisons with the R emmeans package (Lenth et al., 2022). For all statistical analysis, α < 0.05 (rounded to the closest hundredths or first non-zero number thereafter) was used as a threshold for significance.

We used LMMs to determine if percent of dCA1 lesioned impacted home range size, number of territory overlaps with same sex individuals, number of overlaps with opposite sex individuals, number of overlaps with both sexes, number of mating partners, number of pups sired, RER_PRIMARY_, and RER_SECONDARY_. We also included the field enclosure and animal ID (where applicable) as random effects in our models. Figures were generated using GraphPad Prism 10.0.2 (GraphPad Software, San Diego, CA).

The inter-dependency of the measured variables (home range size, overlap with males, overlap with females, overlap with both sexes, number of mating partners, number of pups sired, RER_PRIMARY_, and RER_SECONDARY_) was screened through a principal component analysis (PCA) because our hypotheses were based on the assumption that male prairie vole space use and mating tactics can be influenced by the dorsal HPC (dCA1). This PCA was used to confirm if the associated features of space use and reproductive success were overlapping and if dCA1 lesions caused males to adopt different mating tactics.

## Results

### Subjects

We included all animals possible depending on the nature of the analysis, and sample sizes for each analysis are provided accordingly. Unfortunately, as is common in outdoor semi-natural enclosure studies, some animals lost their radio-collars, or died during the 18 days of radiotracking or prior to recapture and tissue collection. Thus, we were unable to validate surgery treatment and parentage assignment for some animals. Additionally, experimenter error in notetaking of mother-fetus relatedness and inconclusive maternity assignments from CERVUS analyses led to the removal of 2 enclosures from our analyses. Therefore, we were forced to exclude a total of 32 males and 43 females and their fetuses from the final dataset.

We determined, *a priori* based on previous work (Ophir et al., 2008a), that we needed a minimum of 28 fixes from an individual to create accurate home ranges. Fortunately, we were able to reach and exceed this criterion for most of the animals in our experiment. We collected appoximately 34 to 36 fixes per male over the course of the 3-week tracking period.

### dCA1 lesions and the proportion of residents and wanderers, and breeding success

Overall, we recovered 42 residents and 16 wanderers, indicating that residents significantly outnumbered wanderers (Sign Test, two-tailed; *p* < 0.001), which is consistent with previous reports. However, the proportion of the 36 sham and 22 lesioned males that exhibited a resident or wanderer mating tactic was not significantly different (Fisher’s exact test: *p* = 1.00; Figure 2B). Indeed, the ratio of residents to wanderers among lesioned animals was 2.67 (73.0% residents, 27.0% wanderers), and 2.60 (72.2% residents, 27.8% wanderers) among sham animals. Moreover, surgery treatment or mating tactic did not impact the number of mating partners [LMM: surgery × mating tactic *F*_(1,56.45)_ = 0.04, *p* = 0.84; surgery *F*_(1,57.94)_ = 0.01, *p* = 0.92; mating tactic *F*_(1,55.91)_ = 0.39, *p* = 0.54].

Lesioned animals had an average of 57.6% of their dCA1 lesioned (ranging from 25.0 to 93.6%). We saw no lesion outside of the dCA1 region (Figure 2A) and one subject was excluded from analysis due to having a lesion <25%. Notably, in a separate set of animals, we confirmed that dCA1 lesions did not interfere with a male’s ability to form a pair bond (see Supplementary Figure S1 and Supplementary Table S1). Therefore, any differences between residents or wanderers could be attributed to the dCA1’s role in shaping behaviors other than partner preference formation and pair bonding.

### dCA1 lesions and home range size

**Figure 3:**
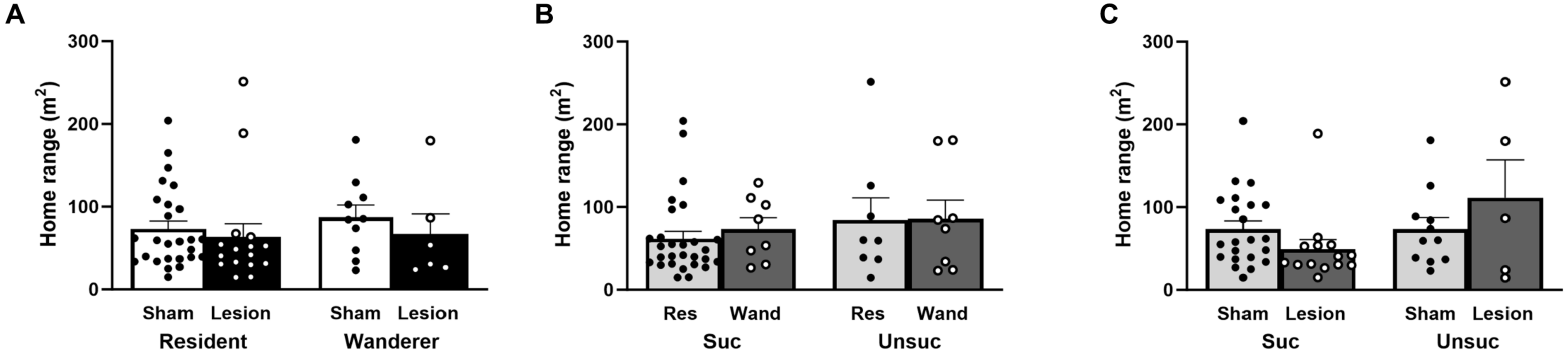
Male home range size in response to surgery treatment, mating tactic, and reproductive success. **(A)** Surgery and mating tactic; dots represent individual data for sham (black solid dots in white bars) and lesioned (black hollow dots in black bars) males. **(B)** Mating tactic and reproductive success; dots represent individual data for resident (black solid dots in light gray bars) and wanderer (black hollow dots in dark gray bars) males. **(C)** Surgery and reproductive success; dots represent individual data for sham (black solid dots in light gray bars) and lesioned (black hollow dots in dark gray bars) males. Data presented as mean ± SEM.

We used home range size (in square meters) as a measure of space use and compared the effects of surgery treatment (sham vs. lesion), mating tactic (resident vs. wanderer), and reproductive success (successful vs. unsuccessful at siring pups). Our three factor LMM showed a nonsignificant trend of reproductive success as a main effect [reproductive success: *F*_(1,52)_ = 3.45, *p* = 0.07], with successfully breeding males tending to have smaller home ranges than unsuccessful males. However, the main effects of surgery and mating tactic were clearly not significant [surgery: *F*_(1,52)_ = 0.02, *p* = 0.89; mating tactic: *F*_(1,52)_ = 0.04, *p* = 0.84]. Two-way interactions between surgery treatment and mating tactic, and between mating tactic and reproductive success did not account for differences in home range size [surgery × mating tactic: *F*_(1,52)_ = 1.82, *p* = 0.18; mating tactic x reproductive success: *F*_(1,52)_ = 0.30, *p* = 0.59; Figures 3A,B and Supplementary Table S1]. Notably, the interaction between surgery and reproductive success was significant [surgery × reproductive success: *F*_(1,52)_ = 5.28, *p* = 0.03; Figure 3C and Supplementary Table S1]. The three-way interaction also did not account for differences in home range size [surgery × mating tactic × reproductive success: *F*_(1,52)_ = 0.003, *p* = 0.96; Supplementary Table S1]. Although no *post hoc* comparisons approached significance, the pattern found within the significant interaction between reproductive success and surgery was that sham males that breed successfully or unsuccessfully had comparably sized home ranges, whereas lesioning males tended to reduce the home range size for successfully reproducing males and increase the home ranges of unsuccessfully reproducing males.

### dCA1 lesions and home range overlap

We investigated the number of home ranges of other conspecifics that subject males overlapped. This provides an estimate of the degree to which males could potentially interact with (or deterred) other males (male–male overlaps) and females (male–female). In male–female overlaps, we excluded the partners of resident males because this is the female with whom a paired male is expected to overlap, by definition. Therefore, male–female overlaps provided an estimate of the degree to which resident males potentially interacted with non-partners (i.e., opportunities for extra pair mating, or ‘roving’).

**Figure 4:**
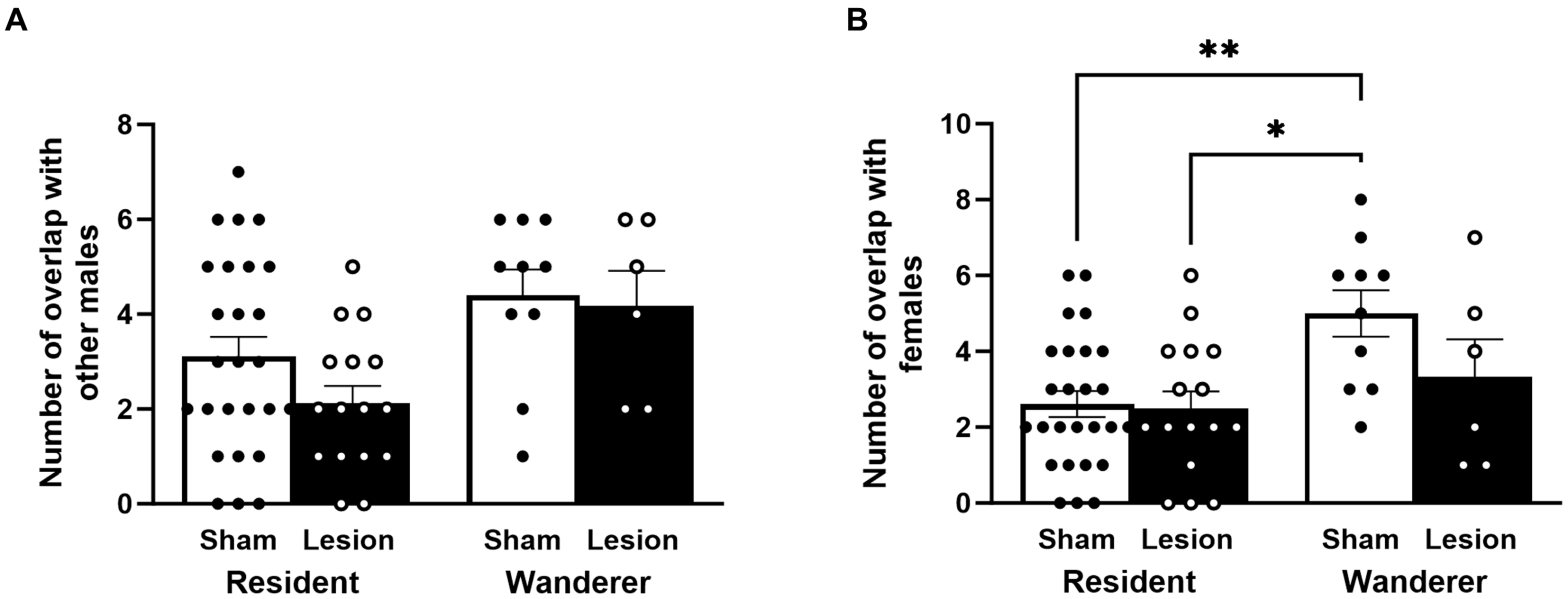
Male patterns of home range overlap. Number of male home ranges overlapped with **(A)** other males and **(B)** with females. Dots represent individual data for sham (black solid dots in white bars) and lesioned (black hollow dots in black bars) males. Data presented as mean ± SEM. *p < 0.05; **p < 0.01.

Focusing specifically on male–male home range overlap, we found that resident male home ranges overlapped with other males less than home ranges of wanderer males [LMM: mating tactic *F*_(1,57.78)_ = 6.07, *p* = 0.02; Figure 4A and Supplementary Table S1]. However, dCA1 lesions did not impact the number of home range overlaps by other males [surgery: *F*_(1,56.26)_ = 0.66, *p* = 0.42], and the interaction between the surgical treatment and mating tactic was not significant [*F*_(1,53.46)_ = 0.40, *p* = 0.53]. *Post hoc* analyses indicated that lesioned residents tended to have fewer home range overlaps with other males than lesion wanderers [*t*_(60.6)_ = −1.87, *p* = 0.07], but this trend was not statistically significant. No other *post hoc* comparisons approached significance.

We examined the number of female home ranges overlapped by males. Once again, resident male home ranges overlapped with females less than home ranges of wanderers [*F*_(1,58)_ = 8.71, *p* = 0.004; Figure 4B and Supplementary Table S1]. Moreover, like male–male home range overlap, neither the surgical treatment nor the interaction between surgical treatment and mating tactic were significantly different for male–female home range overlap [surgery: *F*_(1,58)_ = 2.67, *p* = 0.11; surgery × mating tactic: *F*_(1,58)_ = 2.025, *p* = 0.16]. However, unlike male–male home range overlap, the *post hoc* analyses indicated that sham wanderers overlapped with more females than lesioned residents [*t*_(51.5)_ = −3.04, *p* = 0.02] and sham residents [*t*_(61.9)_ = −3.34, *p* = 0.008]. Notably, lesioned wanderers were no different from residents (lesion or sham) in how many females their home ranges overlapped [lesioned wanderers vs. lesion residents: *t*_(62.5)_ = −0.89, *p* = 0.81; lesioned wanderers vs. sham residents: *t*_(59.8)_ = −0.84, *p* = 0.84]. These data indicate that lesioning the dCA1 of wanderers appears to have caused them to show a pattern of female home range overlap more like residents, but residents were not affected by dCA1 lesions in this way.

### dCA1 lesions and social fidelity

An individual’s RER estimates the probability of two individuals encountering each other based on the proportion of home range they share, given all other individuals in an enclosure. Thus, the RER for a particular animal can be used as a continuous measure of socio-spatial fidelity with a given individual. We therefore recorded the RER that each male exhibited with his most-encountered female (RER_PRIMARY_) and with his second most-encountered female (RER_SECONDARY_). Residents, by definition, have an RER with a partner that is ≥0.5 (and vice versa), whereas wanderers either have an RER with all females that are less than 0.5, or have an RER with a female that is ≥0.5 but the female does not have an RER with that male that is ≥0.5. Therefore, it is expected that residents should have significantly larger RER_PRIMARY_ than wanderers. Similarly, the difference between the RER_PRIMARY_ and RER_SECONDARY_ should be more pronounced for residents than wanderers and therefore, wanderers should have greater RER_SECONDARY_ than residents.

**Figure 5:**
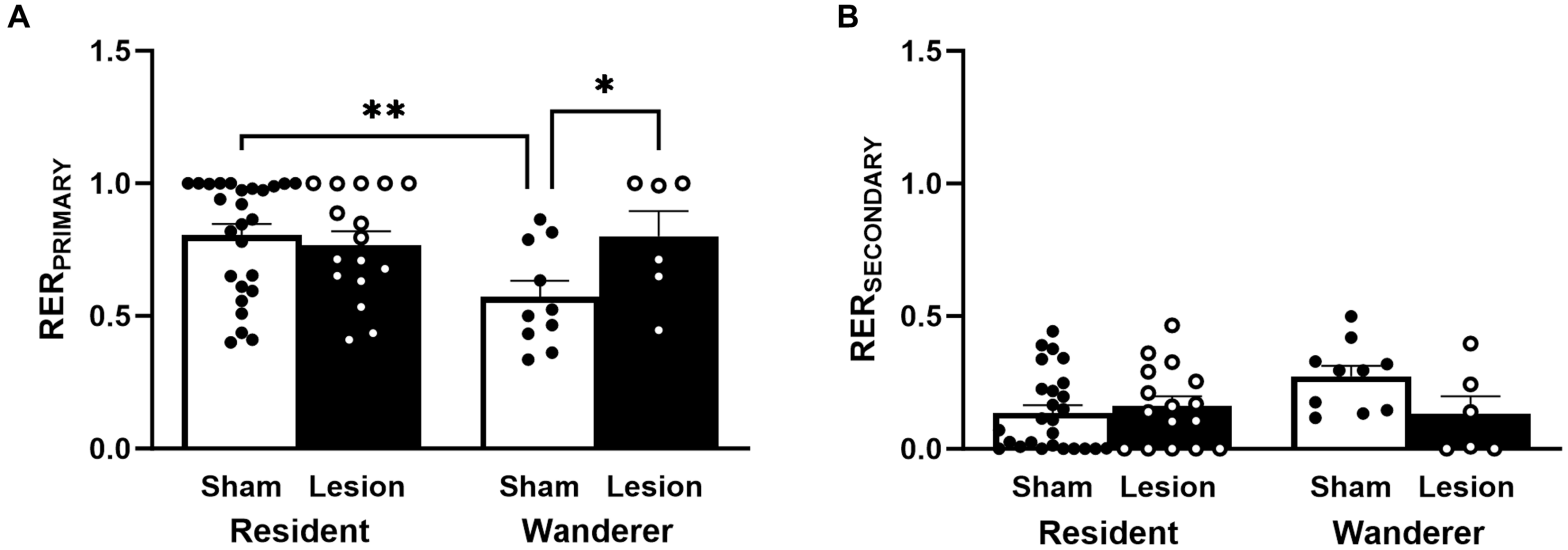
Continuous measure of male social fidelity. **(A)** RER_PRIMARY_ and **(B)** RER_SECONDARY_. Dots represent individual data for sham (black solid dots in white bars) and lesioned (black hollow dots in black bars) males. Data presented as mean ± SEM. *p < 0.05; **p < 0.01.

Neither the main effect of surgical treatment [*F*_(1,58)_ = 2.44, *p* = 0.12] nor mating tactic [*F*_(1,58)_ = 2.65, *p* = 0.11; Figure 5A and Supplementary Table S1] were statistically significant for RER_PRIMARY_. However, the interaction between these factors for RER_PRIMARY_ was statistically significant [*F*_(1,58)_ = 4.59, *p* = 0.04]. Our *post hoc* analyses indicated that, as expected, sham wanderers had significantly lower RER_PRIMARY_ than sham residents [*t*_(62.3)_ = 2.86, *p* = 0.006]. However, lesion wanderers had significantly larger RER_PRIMARY_ than sham wanderers [*t*_(61.7)_ = 2.04, *p* < 0.05]. Sham and lesion residents did not differ in their RER_PRIMARY_ [*t*_(54.8)_ = −0.50, *p* = 0.62]. These data indicate that lesioned dCA1 did not impact this metric among residents, but it did shift lesion wanderer RER_PRIMARY_ to resemble residents (mean RER_PRIMARY_: sham wanderer 0.57; lesion wanderer 0.80; sham resident 0.80; lesion resident 0.77).

Like the results for RER_PRIMARY_, we found no main effects for RER_SECONDARY_ [surgery treatment: *F*_(1,58)_ = 1.86, *p* = 0.18; mating tactic: *F*_(1,58)_ = 1.58, *p* = 0.21; Figure 5B and Supplementary Table S1]. However, RER_SECONDARY_ did show a significant interaction between surgery treatment and mating tactic [*F*_(1,58)_ = 3.93, *p* = 0.05]. *Post hoc* comparisons showed that sham wanderers tended to have higher RER_SECONDARY_ than sham residents [*t*_(62.3)_ = −2.46, *p* = 0.08], but this trend was not statistically significant. No other *post hoc* comparisons approached significance.

### dCA1 lesions and successful fertilizations

**Figure 6:**
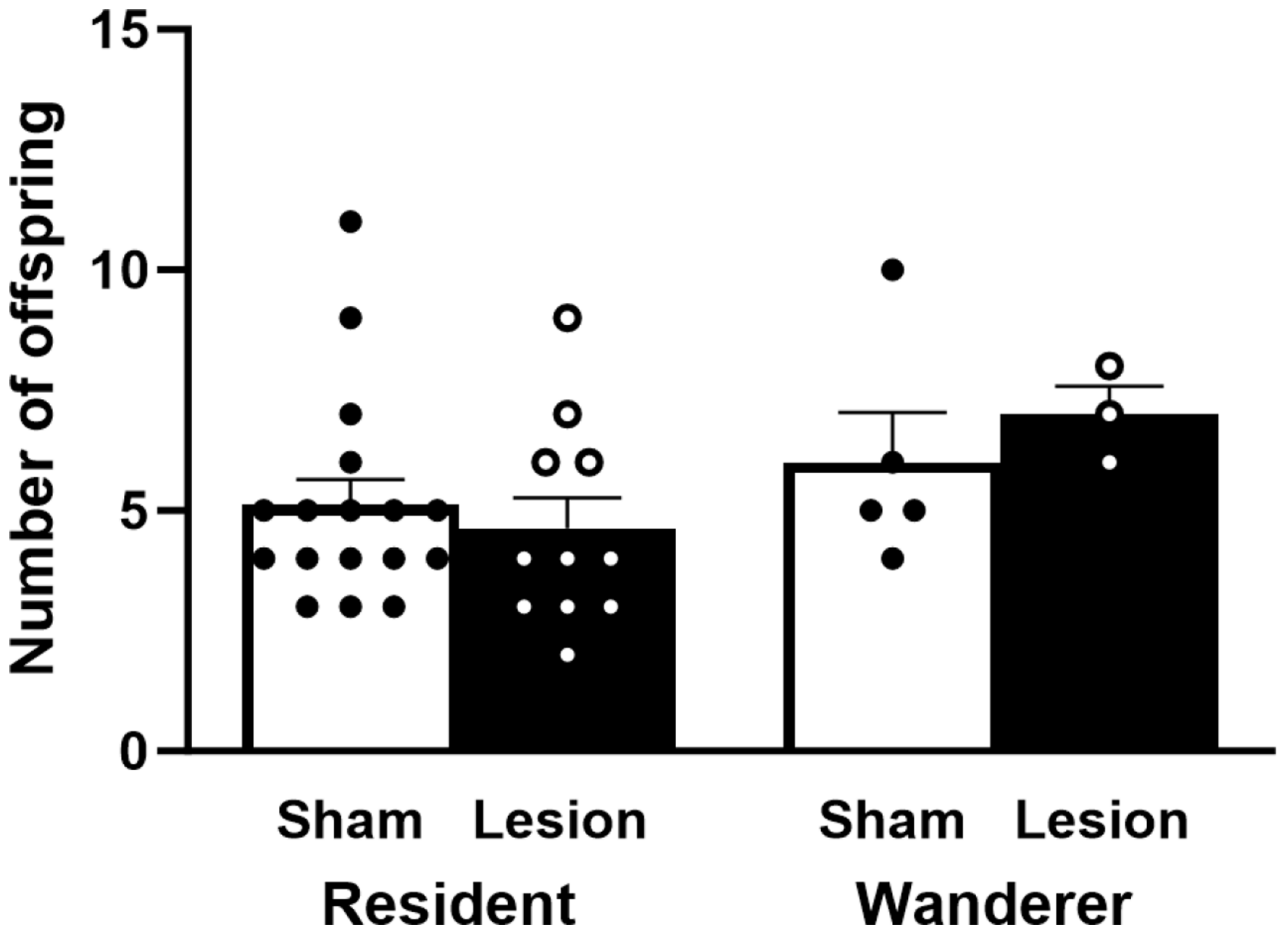
Number of successfully sired offspring. Reproductively unsuccessful males were not included in this analysis. Dots represent individual data for sham (black solid dots in white bars) and lesioned (black hollow dots in black bars) males. Data presented as mean ± SEM.

To determine if reproductive success was impacted by dCA1 lesions, we compared the number of fertilized embryos across mating tactic and surgical treatments. Our results showed that reproductive success did not differ across surgical treatment [*F*_(1,58)_ = 0.03, *p* = 0.85] or mating tactic [*F*_(1,58)_ = 0.0003, *p* = 0.99], and there was no interaction effect between them [*F*_(1,58)_ = 0.13, *p* = 0.72; Figure 6 and Supplementary Table S1].

Although the number of offspring sired did not appear to differ across factors, we asked whether dCA1 affected the sexual fidelity of male residents, or the likelihood of being cuckolded. Out of the 33 resident pairs with reproductive success (Table 2), we found that 63.6% of the couples (*n* = 21) produced viable embryos with only their partners (i.e., ‘true residents’ that engaged exclusively in intra-pair fertilizations; IPF). On the other hand, 27.3% (*n* = 9) of the couples had a female that mated with at least one other male that was not her partner (i.e., male cuckolds), whereas 9.1% (*n* = 3) of the couples had a male that fertilized offspring with a female other than his partner (i.e., roving residents). Thus, in total, 36.4% (*n* = 12) of the couples had at least one member of the pair that produced an extra-pair fertilization (EPF). Overall, there was no difference in the proportion of IPFs between sham and lesion pairs (Sign Test, two-tailed; *p* = 0.52), the overall proportion of EPFs (Sign Test, two-tailed; *p* = 0.39), or the proportion of EPFs among cuckolded males (Sign Test, two-tailed; *p* = 0.51) or roving males (Sign Test, two-tailed; *p* = 0.10). Taken together, dCA1 lesions did not affect the reproductive success within tactics or sub-tactic within resident males.

**Table 2:** Distribution of resident pair sexual monogamy.

	Male surgery treatment	
**Resident pairs**	Sham	Lesion
Male IPF - Female IPF	12	9
Male IPF - Female EPF	6	3
Male EPF - Female IPF	2	1

IPF, intra-pair fertilization; EPF, extra-pair fertilizations.

### Principal component analysis of the impact of dCA1 lesions

**Figure 7:**
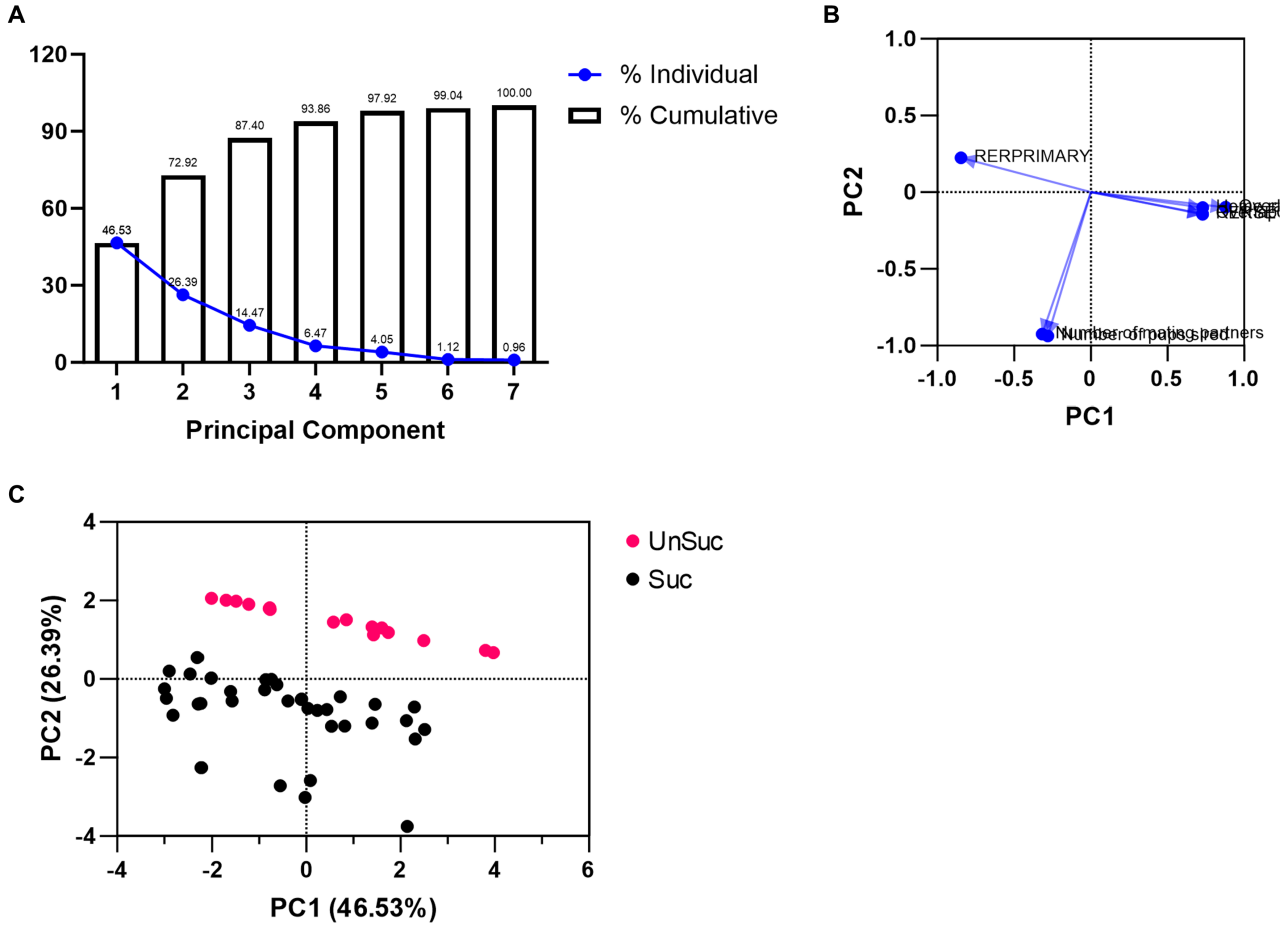
PCA plots. **(A)** Individual (blue solid dots with blue solid lines) and cumulative (bar graphs) proportion of variance for each PC. **(B)** Variable loadings for PC1 and PC2. **(C)** PC1 and PC2 scores for each reproductively successful (black dots) and reproductively unsuccessful (red dots) male.

Our principal component analysis (PCA) included home range size, overlap with males, overlap with females, number of mating partners, number of pups sired, RER_PRIMARY_, and RER_SECONDARY_. The outcome produced two orthogonal principal components (PC) that explained 72.92% of all data (Figure 7A and Supplementary Table S2). The first PC (46.53%) was informed by 5 of the 7 factors that had loadings of 0.4 or greater (Stevens, 1992; Figure 7B and Supplementary Table S2). These factors were home range size (0.728), RER_PRIMARY_ (−0.847), RER_SECONDARY_ (0.728), male–male home range overlaps (0.727), and male–female home range overlap (0.878; Supplementary Table S3). The second PC explained an additional 26.39% of the variance, but only had two loadings that were greater than 0.4 (number of mating partners [−0.926], and number of sired offspring [−0.938]; Supplementary Table S3), and should therefore be considered falling short of practical significance (Stevens, 1992). Considering the factors that contributed most to each of these PCs, we interpreted PC1 as describing “mating tactics” and PC2 as describing “reproductive success.” Despite the low number of factor loadings for PC2, plotting the PC1 and PC2 scores of each male showed a distinct divide between males that were reproductively successful and those that were not (Figure 7C).

**Figure 8:**
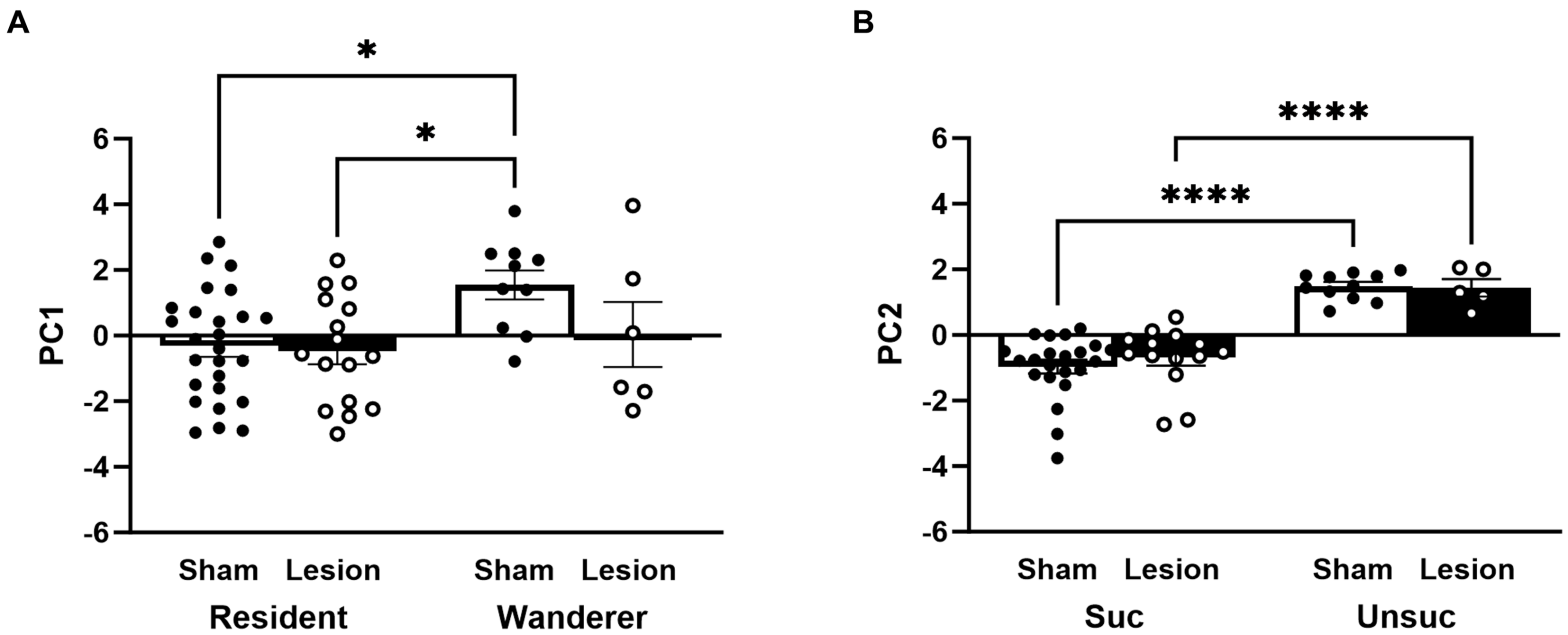
PC1 and PC2 scores stratified by surgery treatment, mating tactic, and reproductive success. **(A)** Impact of surgery treatment and mating tactic on PC1 scores. **(B)** Impact of surgery treatment and reproductive success on PC2 scores. Dots represent individual data for sham (black solid dots in white bars) and lesioned (black hollow dots in black bars) males. Data presented as mean ± SEM. *p < 0.05; ****p < 0.0001.

We compared each PC as a factor using a LMM. PC1 revealed a significant main effect of mating tactic [*F*_(1,54)_ = 7.34, *p* = 0.009; Figure 8A and Supplementary Table S1] but not a main effect of surgical treatment [*F*_(1,54)_ = 1.31, *p* = 0.26]. The interaction between surgical treatment and mating tactic was not significant [*F*_(1,54)_ = 1.76, *p* = 0.19]. Notably, the *post hoc* analyses demonstrated that sham wanderers had significantly higher PC1 scores than both sham residents [*t*_(54)_ = −2.95, *p* = 0.02] and lesioned residents [*t*_(54)_ = −2.94, *p* = 0.02]. However, lesioned wanderers were no different from either group of residents [*vs* sham residents: *t*_(54)_ = 0.46, *p* = 0.97; vs. lesion residents: *t*_(54)_ = −0.62, *p* = 0.93]. These data are consistent with the individual measures reported above that indicate that lesions of the dCA1 do not impact space use among residents but appear to shift wanderer space use toward the resident-typical pattern. For PC2, our LMM analysis revealed a significant main effect of reproductive success [*F*_(1,48)_ = 84.79, *p* < 0.00001; Figure 8B and Supplementary Table S1], but no main effect of surgical treatment [*F*_(1,48)_ = 0.01, *p* = 0.92] or interaction [*F*_(1,48)_ = 0.37, *p* = 0.55]. *Post hoc* comparisons showed that males that reproduced had lower PC2 scores than those that did not reproduce [sham: *t*_(48)_ = −7.86, *p* < 0.0001; lesion: *t*_(48)_ = −4.83, *p* = p < 0.0001].

## Discussion

We leveraged the natural variation among male prairie voles to determine how and if the dCA1 region of the HPC influenced or altered monogamous (resident) and non-monogamous (wanderer) mating tactics. To this end, we examined the effects of targeted lesions of the dCA1 to investigate the role of presumed impaired spatial cognition on mating tactics among male prairie voles living in naturalistic conditions. We assumed that resident males potentially rely on spatial cognition primarily for territorial and mate guarding purposes. We also assumed that wandering males potentially rely on spatial cognition for enhanced spatial navigation of large areas and enhanced navigation of the socio-spatial landscape to maximize the number of potential mates. We found that the patterns of space use by male wanderers in this highly complex environment was influenced by dCA1 lesions, such that lesioned wanderers appeared to use space much like resident males. Notably, resident males were not impacted by dCA1 lesions in any of the measures we were able to collect. These results raise the curious possibility that resident mating tactics among male prairie voles are not dependent on a functional hippocampus, whereas the patterns of space use associated with ‘typical’ wandering behavior do rely on the dCA1.

### dCA1 lesions unexpectedly did not impact home range size

Considering that our lesions were restricted to the dCA1 of the HPC and that they did not appear to impact pair bonding behavior (Supplementary Figure S1 and Supplementary Table S1), we anticipated the most observable effects of our lesions would be found on how animals used space. In particular, we expected to observe gross changes in home range size to result from dCA1 lesions. Strangely, mean home range sizes were identical between residents and wanderers, whether dCA1 was lesioned or not. This is particularly surprising because the home range sizes of residents are usually smaller than those of wanderers, even if the mean difference is not significant (Solomon and Jacquot, 2002; Ophir et al., 2007, 2008c, 2012; Zheng et al., 2013; Blondel et al., 2016; Rice et al., 2022). One possible explanation for these uncharacteristic results is that the lesioned animals altered their space use in a way that impacted the behavior of the sham animals. Indeed, the social composition of animals within an enclosure can alter spatial cognition (Rice et al., 2019). However, in this instance, we believe this outcome might relate more to the population density that we created. In most of the previous work using enclosures of this size, the number of animals introduced has been 6 males and 6 females (or 0.02 voles/m^2^). However, to ensure the number of lesioned and sham animals per enclosure was sufficiently large to compare, the number of animals we introduced to each enclosure in this study was 10 males and 10 females (or 0.033 voles/m^2^). Thus, the population density of the enclosures in this study was 66.5% greater than most studies of this kind, which could understandably limit size of home ranges and reduce the variance within. Although some attention has been directed to examining the influence of population density (Blondel et al., 2016), more work is clearly needed to understand how this impacts the social dynamics and reproductive decision making within populations.

Notably, the one significant relationship we found with home range size was the interaction between reproductive success and surgery. In this case, successfully and unsuccessfully reproducing males showed comparably sized home ranges if their dCA1 was intact (sham), whereas lesioning the dCA1 appeared to shrink the home range sizes of successfully breeding males and increase the home range size of unsuccessfully breeding males overall. Thus, to the extent that home range size and reproductive success are linked, perhaps dCA1 lesions make it more difficult for males to acquire mates and breed while also maintaining a relatively large home range. In other words, the cognitive challenges of balancing the demands of monitoring a large territory and locating potential mates may be too much for animals with non-functional dCA1s.

### dCA1 lesions did not alter the proportion of residents and wanderers

Because the HPC, and the dCA1 specifically, is so critical for spatial cognition (Tsien et al., 1996; Moser and Moser, 1998; Fanselow and Dong, 2010), and because home range size is inherently based on space use, we predicted that dCA1 lesions should impact the probability of males adopting either resident or wandering behaviors. Specifically, we anticipated a shift in the overall proportion of residents to wanderers or vice versa. However, the proportion of residents to wanderers (with or without dCA1 lesions) were consistent with the proportions that have been reported in numerous studies (see Madrid et al., 2020). In fact, the ratio of residents to wanderers among lesioned animals and sham animals was nearly identical. Therefore, the proportion of residents to wanderers in a population does not appear to be under the influence of the dCA1, and the factors that lead to adopting a particular tactic may not be directly tied to processing spatial information exclusively. This does not rule out the possibility that the likelihood of becoming a resident or wanderer is influenced by spatial cognition, *per se*. For instance, it is entirely possible that processing social identity and/or contextually linking that information to a particular location in space could impact the decision to adopt a particular tactic (Phelps and Ophir, 2009; Ophir, 2017). Similarly, it is possible that the social context (i.e., the composition of males and females engaged in different mating tactics) could drive the proportion of animals that adopt each tactic (Rice et al., 2019) independently of spatial processing.

### dCA1 lesions consistently impacted wanderer space use

It is important to acknowledge that the populational ratio of the resident and wandering tactics ignores the variation within and between tactics at the level of the individuals. In other words, even if dCA1 function does not appear to directly impact the total probability of becoming either a resident or a wanderer, it might impact the efficacy with which some animals are able to optimally perform within a given tactic. Therefore, we predicted that lesions of the dCA1 would either (1) shift residency behavior to resemble the typical ‘wanderer’ phenotype by increasing home range size, increasing the number of home range overlaps, and possibly impacting reproductive success if HPC function promotes residency behavior (see below), or (2) shift wandering behavior to resemble the typical ‘resident’ phenotype by decreasing home range size, decreasing the number of home range overlaps, and possibly impacting reproductive success if HPC function promotes wandering behavior.

Unlike the size of home ranges, many other measures of space use showed a consistent pattern where lesioned, but not intact, wanderers resembled residents. For example, sham wanderers had numerous home range overlaps with females, which is the typical pattern for wanderers. In contrast, dCA1 lesioned wanderers had fewer home range overlaps with females, at frequencies that were comparable to both lesioned and sham resident males; both lesioned and sham residents had comparably low numbers of home range overlaps with (non-partner) females. The fact that this was not the case for male–male overlaps highlights the point that space use relative to females (and the potential acquisition of mates) was particularly sensitive to dCA1 lesions among wanderers. Similarly, we compared the RERs for the female that overlapped each males’ home range the most (the RER_PRIMARY_), and once again demonstrated that lesioned wanderers shifted their patterns of space use to resemble the typical resident pattern of behavior (i.e., relatively large), whereas sham wanderers showed the RER_PRIMARY_ pattern that is typically associated with wandering (i.e., relatively small). Furthermore, although this effect fell just short of statistical significance, the general patterns showed that dCA1 lesioned wanderers also resembled residents more than sham wanderers for the second most overlapped female (RER_SECONDARY_). Recall that RERs characterize the proportion of home range space a given male overlapped a given female, relative to all other females. Therefore, a RER represents the probability that a male was likely to encounter (and presumably interact with) a particular female. Taken together, residents and lesioned wanderers had a much higher probability of interacting with just one female (high RER_PRIMARY_ and low RER_SECONDARY_), whereas sham wanderers showed relatively low RER_PRIMARY_ and high RER_SECONDARY_, indicating they were more likely to encounter multiple females with similar probabilities. Finally, the PCA analysis that was informed by metrics of space use (PC1) also demonstrated this pattern of dCA1 lesioned wanderers being more like residents than sham wanderers. Overall, these data are highly consistent with each other and support the hypothesis outlined above that wandering males rely on dCA1-dependent spatial cognition more than resident males do, possibly for enhanced spatial navigation of large areas and enhanced navigation of the socio-spatial landscape.

The fact that wanderers were particularly sensitive to the dCA1 manipulation is not without precedent. Indeed, a comparison of two closely related species of mice with different mating systems demonstrated a greater capacity for spatial cognition among the polygynous species compared to the monogamous species (Jasarevic et al., 2012). Furthermore, Ophir (2017) suggested that modulation of spatial cognition by oxytocin and vasopressin signaling within the canonical spatial cognition neural circuit (i.e., the HPC-anterior thalamus [AT]-retrosplenial cortex [RSC] axis) might shape mating tactics or predispose some animals to excel at a particular mating tactic. Indeed, oxytocin and vasopressin have long been implicated in the modulation of learning and memory (Mcewen, 2004). Notably, the differences in oxytocin receptors within the HPC (Ophir et al., 2012) and vasopressin receptors within the lateral-dorsal subunit of the AT and the RSC (Ophir et al., 2008c) not only predict the reproductive success among residents and wanderers, they also strongly differ between reproductively successful and unsuccessful wanderers. Thus, the natural variation in nonapeptide receptor density (and therefore signaling and neuromodulation within much of the spatial cognition axis) among wanderers is highly consistent with the artificial variation that the lesions of the dCA1 appear to have imposed. Whether lesions of the dCA1 induced a disruption of spatial cognition that impacted wandering males’ ability to remember the location of potential mates, the location of resources and nesting sites, or other types of important information that are based in space remains an open question. Indeed, perhaps the dCA1 lesion made it prohibitive for wanderers to track multiple females using allocentric information, such that lesioned wanderers were more reliant on local landmark cues to navigate their space. This explanation assumes wanderers use this strategy to actively track multiple females, which may or may not be the case (Gaulin, 1992; Phelps and Ophir, 2009). Nevertheless, we suspect that lesions of the dCA1 eliminated neurons (including neurons sensitive to oxytocin) that wanderers might preferentially rely on in important ways to move through their world.

Moreover, Rice et al. (2022) found that after living in semi-natural field enclosures, the natural variation in space use among wanderers, but not residents, predicted performance in the Morris water maze task in the lab. These data indicated that spatial cognition (learning in this case) was strongly associated with the degree to which wanderers demonstrated ‘typical’ patterns of wandering behavior (i.e., large home ranges overlapping those of many other conspecifics) or patterns of space use more like residents. Stepping back, the picture that appears to emerge from these studies and the results of the current investigation is that patterns of space use closely associated with stereotypical wandering behavior is particularly sensitive to spatial cognition and the successful function of the HPC-AT-RSC axis.

### Resident patterns of space use were unaffected by dCA1 lesions

We were surprised to find that resident prairie voles’ space use behavior was unaffected by dCA1 HPC lesions. As such, we conclude that the cognitive challenges that impact how they use space are independent of dCA1 function. Nevertheless, monogamous behavior must leverage several forms of social and spatial cognition to defend against potential cuckoldry and resource theft, and to participate in mating opportunities with nearby ovulating females (Brotherton and Komers, 2003). Thus, different mating tactics presumably are associated with different selective pressures to integrate spatial information (i.e., home range location and boundaries; location of conspecifics) with social information (i.e., sex, identity, partner status, previous positive or agonistic encounters) and account for the spatial distribution of specific social partners (Ophir, 2017; Rice et al., 2017). Although dCA1 lesions did not appear to influence how residents used space, we do not conclude that residents do not rely on spatial cognition at all. Rather, we suspect that the ways in which they use and process space differ from the ways wanderers process this information. Indeed, other regions of the HPC (e.g., CA2, CA3, and dentate gyrus subregions) and other components of the HPC-AT-RSC axis might alter the spatial cognition of residents in ways that would affect patterns of space use. Continued work to probe what aspects of spatial cognition and their underlying neural regions of control impact residents and wanderers differently will reveal much about how selection has shaped mating tactics and the ability of some individuals to excel at some tactics better than others.

### Fertilization success was unaffected by dCA1 lesions

Finally, our results also investigated how dCA1 functioning impacted the reproductive success (i.e., number of fertilized embryos) of males that had adopted each mating tactic. Previous work has shown that resident males tend to fertilize more embryos than wanderers (Ophir et al., 2008b). In this study, we did not replicate this result. Unlike our study that tracked animals for one reproductive cycle, Shuster et al. (2019) showed the reproductive success of wanderers tended to equal that of residents over several reproductive cycles. This difference in reproductive output has largely been attributed to the assumption that lifetime reproductive success should be balanced among mating tactics for selection to maintain both phenotypes (Rice et al., 2019; Shuster et al., 2019). It is unclear if the dCA1 lesions erased this difference indirectly by altering measures of social dynamics we were unable to detect. However, the data did not show a systematic advantage or disadvantage of lesioned residents or wanderers in comparison to sham residents or wanderers. It is also possible that the high population density experienced by animals serving in this experiment, relative to similar previous experiments (see above), might account for the shift away from residents having a relative reproductive advantage. We were also surprised that resident males with dCA1 lesions produced similar numbers of EPFs compared to resident males without dCA1 lesions. However, considering that the results demonstrated that dCA1 lesions did not impact residents, perhaps this should not be surprising. Indeed, this is just one more instance in which resident males seemed unaffected by the consequences of dCA1 lesions, underscoring the point that the behaviors and forms of spatial cognition that residents rely on are unlikely to be those that are influenced by dCA1 functioning.

## Conclusion

Our results showed that the effects of dCA1 lesions are dependent on the mating tactic individual males adopted in a semi-naturalistic setting. Specifically, dCA1 lesions altered the home range size of males that bred successfully. More generally, dCA1 lesions altered the patterns of space use of wanderer males to resemble patterns comparable to resident males, whereas we measured no difference in territory size or patterns of space use between lesioned and sham residents. In other words, we saw a breakdown in male space use among wanderers, but not residents. These results support the hypothesis that the wanderer mating tactic relies, in part, on HPC (dCA1) processing. Moreover, these data are consistent with neuroanatomical data on nonapeptide receptor density and behavioral data linking field behavior with lab measures of spatial cognition that indicate that variation in mating tactics is particularly observable within the phenotypic variation expressed by wanderers. Stepping back, our results provide insights into the ways spatial cognition, and the mechanisms that govern it, shape mating tactics. Indeed, it is likely that the coordination of social information with spatial cognition helps shape reproductive decision-making in prairie voles. However, our study suggests that different mating tactics rely on different forms and spatial cognition to succeed at the tasks that face them.

## Data availability statement

The original contributions presented in the study are included in the article/Supplementary materials, further inquiries can be directed to the corresponding author.

## Ethics statement

The animal study was approved by Institutional Animal Care and Use Committee of Cornell University. The study was conducted in accordance with the local legislation and institutional requirements.

## Author contributions

LS: Data curation, Formal analysis, Investigation, Methodology, Visualization, Writing – original draft. CF: Conceptualization, Data curation, Formal analysis, Investigation, Methodology, Supervision, Writing – original draft. PP: Data curation, Investigation, Methodology, Writing – review & editing. SB: Data curation, Formal analysis, Methodology, Validation, Writing – review & editing. AO: Conceptualization, Funding acquisition, Project administration, Resources, Supervision, Visualization, Writing – review & editing.

## Footnotes

^1^https://bitbucket.org/cornell_bioinformatics/amplicon/src/master/
