# Supplementary material for: Dorsal CA1 lesions of the hippocampus impact mating tactics in prairie voles by shifting non-monogamous males’ use of space to resemble monogamous males
Source: Front Behav Neurosci. 2024 Feb 26;18:1355807. doi: 10.3389/fnbeh.2024.1355807 (PMC10925758; doi:10.3389/fnbeh.2024.1355807)
Supplement: Supplementary file 1 [file Data_Sheet_1.docx]

**SUPPLEMENTARY MATERIALS**

**Supplementary Table 1 |** Statistical results of our linear mixed models.

| **Dependent variable** | **Source** | **df** | ***F*** | ***p*** |
| --- | --- | --- | --- | --- |
| Home range size (m^2^) (Figure 2 A-C) | Surgery | 1,52 | 0.02 | 0.89 |
|  | Mating tactic | 1,52 | 0.039 | 0.84 |
|  | Reproductive success | 1,52 | 3.449 | *0.07* |
|  | Surgery x mating tactic | 1,52 | 1.819 | 0.18 |
|  | Surgery x rep. suc. | 1,52 | 5.284 | ***0.03*** |
|  | Mating tactic x rep. suc. | 1,52 | 0.302 | 0.59 |
|  | 3-way interaction | 1,52 | 0.0003 | 0.96 |
| Male-male home range overlap (Figure 3A) | Surgery | 1,56.26 | 0.662 | 0.42 |
|  | Mating tactic | 1,57.78 | 6.067 | ***0.02*** |
|  | Interaction | 1,53.46 | 0.402 | 0.53 |
| Male-female home range overlap (Figure 3B) | Surgery | 1,58 | 2.672 | 0.11 |
|  | Mating tactic | 1,58 | 8.714 | ***0.004*** |
|  | Interaction | 1,58 | 2.025 | 0.16 |
| RER_PRIMARY_ (Figure 4A) | Surgery | 1,58 | 2.440 | 0.12 |
|  | Mating tactic | 1,58 | 2.649 | 0.11 |
|  | Interaction | 1,58 | 4.591 | ***0.04*** |
| RER_SECONDARY_ (Figure 4B) | Surgery | 1,58 | 1.863 | 0.18 |
|  | Mating tactic | 1,58 | 1.583 | 0.21 |
|  | Interaction | 1,58 | 3.932 | ***0.05*** |
| Number of offspring (Figure 5) | Surgery | 1,58 | 0.034 | 0.85 |
|  | Mating tactic | 1,58 | 0.0003 | 0.99 |
|  | Interaction | 1,58 | 0.128 | 0.72 |
| PC1 (Figure 7A) | Surgery | 1,54 | 1.309 | 0.26 |
|  | Mating tactic | 1,54 | 7.337 | ***0.009*** |
|  | Interaction | 1,54 | 1.757 | 0.19 |
| PC2 (Figure 7B) | Surgery | 1,48 | 0.0095 | 0.92 |
|  | Reproductive success | 1,48 | 84.791 | ***3.51E-12*** |
|  | Interaction | 1,48 | 0.367 | 0.55 |
| PPT Chamber Duration (Supp. Figure 1A) | Surgery | 1,34 | 0.0066 | 0.94 |
|  | Chamber | 1,34 | 5.649 | ***0.02*** |
|  | Interaction | 1,34 | 0.4002 | 0.53 |
| PPT Total Contact Duration (Supp. Figure 1B) | Surgery | 1,34 | 0.0360 | 0.85 |
|  | Stimulus | 1,34 | 22.745 | ***3.41E-05*** |
|  | Interaction | 1,34 | 0.0189 | 0.89 |

**Supplementary Table 2 | PCA tabular results.** PC summary of eigen values, proportion of variance, and cumulative proportion of variance for each principal component (PC).

| PC summary | **PC1** | **PC2** | **PC3** | **PC4** | **PC5** | **PC6** | **PC7** |
| --- | --- | --- | --- | --- | --- | --- | --- |
| Eigenvalue | 3.257 | 1.847 | 1.013 | 0.4527 | 0.2837 | 0.07868 | 0.06720 |
| Proportion of variance | 46.53% | 26.39% | 14.47% | 6.47% | 4.05% | 1.12% | 0.96% |
| Cumulative proportion  of variance | 46.53% | 72.92% | 87.40% | 93.86% | 97.92% | 99.04% | 100.00% |

**Supplementary Table 3 | Variable loadings for PC1 and PC2.**

| **Variable** | **PC1** | **PC2** |
| --- | --- | --- |
| Home range size | 0.728 | -0.101 |
| RER_PRIMARY_ | -0.847 | 0.224 |
| Overlap with other males | 0.727 | -0.142 |
| Overlap with females | 0.878 | -0.099 |
| Number of mating partners | -0.318 | -0.926 |
| Number of pups sired | -0.281 | -0.938 |
| RER_SECONDARY_ | 0.728 | -0.145 |

**Supplementary Table 4 | Sample sizes for home range size (Figure 2).**

| **Surgery treatment** | **Mating tactic** | **Reproductive success** | **n** |
| --- | --- | --- | --- |
| Sham | Resident | Successful | 17 |
| Lesion | Resident | Successful | 11 |
| Sham | Wanderer | Successful | 5 |
| Lesion | Wanderer | Successful | 3 |
| Sham | Resident | Unsuccessful | 6 |
| Lesion | Resident | Unsuccessful | 2 |
| Sham | Wanderer | Unsuccessful | 5 |
| Lesion | Wanderer | Unsuccessful | 3 |

**Supplementary Table 5 | Sample sizes for home range overlaps with other males or with females (Figure 3).**

| **Surgery treatment** | **Mating tactic** | **n** |
| --- | --- | --- |
| Sham | Resident | 26 |
| Lesion | Resident | 16 |
| Sham | Wanderer | 10 |
| Lesion | Wanderer | 6 |

**Supplementary Table 6 | Sample sizes for RER_PRIMARY_ and RER_SECONDARY_ (Figure 4).**

| **Surgery treatment** | **Mating tactic** | **n** |
| --- | --- | --- |
| Sham | Resident | 26 |
| Lesion | Resident | 16 |
| Sham | Wanderer | 10 |
| Lesion | Wanderer | 6 |

**Supplementary Table 7 | Sample sizes for number of offspring sired (Figure 5).**

| **Surgery treatment** | **Mating tactic** | **n** |
| --- | --- | --- |
| Sham | Resident | 17 |
| Lesion | Resident | 11 |
| Sham | Wanderer | 5 |
| Lesion | Wanderer | 3 |

**Supplementary Table 8 | Sample sizes for PC1 loadings (Figure 7A).**

| **Surgery treatment** | **Mating tactic** | **n** |
| --- | --- | --- |
| Sham | Resident | 26 |
| Lesion | Resident | 16 |
| Sham | Wanderer | 10 |
| Lesion | Wanderer | 6 |

**Supplementary Table 9 | Sample sizes for PC2 loadings (Figure 7B).**

| **Surgery treatment** | **Mating tactic** | **n** |
| --- | --- | --- |
| Sham | Successful | 22 |
| Lesion | Successful | 14 |
| Sham | Unsuccessful | 11 |
| Lesion | Unsuccessful | 5 |

**Supplementary Table 10 | Sample sizes for partner preference test (Supplementary Figure 1).**

| **Surgery treatment** | **n** |
| --- | --- |
| Sham | 7 |
| Lesion | 10 |

***Partner Preference Test***

In addition to the fieldwork, we also confirmed that dCA1 lesions did not interfere with the ability to form pair bonds. We assessed this by conducting dCA1 lesions as described in the manuscript and then assessing the pair bond in the partner preference test (PPT) in a separate group of males. We used 20 males (sibling pairs, with each pair having one sham and one lesion male), and 25 females (n = 20 partners, n = 5 stimulus females).

*Methods*

Our partner preference tests were run based on the well-established paradigm originally developed by Williams et al., (1992). In brief, we exposed females to soiled bedding from their partner males 48 hours before pairing to induce sexual receptivity. Males were also exposed to the soiled bedding from the female vole with which they would be paired (their ‘partners’). Five stimulus females (‘strangers’) were exposed to soiled bedding from a male (n = 5) randomly chosen from our colony that was not a part of this experiment. Females were used as stimulus for a maximum of four times, and no more than once per day. Male subjects and their female partners were housed together for 24 hours to induce pair bond formation, after which we conducted a PPT.

PPTs were conducted in a rectangular (106.7 x 50.8 x 30.5 cm), opaque, white acrylic apparatus (1.3 cm thick walls) with three chambers, including a central neutral zone (45.7 x 50.8 x 30.5 cm) with two choice chambers (27.9 x 50.8 x 30.5 cm) on either side. Two rectangular cutouts in the opposite walls of the middle chamber allowed passage between the neutral zone and the choice chambers. Females were tethered to a side chamber using a lightweight chain stringer typically used for fishing that was attached to a zip-tie collar placed around the animal’s neck a day before testing. Animals adjust quickly to such tethering, which enables free movement within a chamber (Wolff and Dunlap, 2002, Ophir et al., 2007). The ‘partner’ was tethered to one chamber and the ‘stranger’ was tethered in the opposite chamber.

To initiate the PPT, the male subject was placed in the center of the neutral zone and allowed to freely move throughout the test chambers. We video recorded the PPT with a video camera positioned overhead (Sony Handycam [CX405] Sony, New York City, NY, U.S.A.). We ended the test after 3 hours, and then humanely euthanized males and collected brain tissue for lesion quantification as detailed in the manuscript. Scorers unaware of the treatment conditions analyzed the videos using Observer XT v13 software (Noldus Information Technology, Leesburg, VA, USA). We quantified the amount of time the subject spent in each chamber (partner chamber and stranger chamber) and the amount of time spent in total side-by-side contact with each female.

*Data Analysis*

For the partner preference tests, we used linear mixed models (LMMs) followed by a Type III ANOVA for F-tests based on Satterthwaite’s method to examine how lesions affected pair bond formation as measured by time spent in each chamber and time spent in contact with each female. Animal ID was included as a random effect in our model.

Partner preferences were assessed based on the total time subject males spent in side-by-side contact with each female (Williams et al., 1992). Individual partner preferences are typically defined as instances where subject males spend twice as much time in side-by-side contact with partners compared to time in side-by-side contact with strangers. Specifically, we compared mean times (+ standard error of the mean; SEM) subjects spent in side-by-side contact with each female. Thus pair-bonds were assessed if the group means for time spent in contact with their partner was significantly greater than the time spent in contact with the stranger.

*Results*

Although we began the experiment with 10 lesion males and 10 sham males, brain lesion calculation and a series of technical issues reduced our sample size to 7 sham males and 10 lesioned males. Our model showed that subjects spent significantly more time in the chamber containing the partner stimulus (F_(1, 34)_ = 5.649, *p* = 0.02; **Supplementary Figure 1A** and **Supplementary Table 1**). Surgery treatment or interaction had no effect on chamber duration (surgery: F_(1, 34)_ = 0.0066, *p* = 0.94; surgery x chamber: F_(1, 34)_ = 0.4002, *p* = 0.53). *Post hoc* comparisons showed that lesioned subjects spent more time in the chamber containing their partner than in the chamber containing a stranger (*t*_(19.3)_ = 2.203, *p* = 0.04) but not sham subjects (*t*_(19.3)_ = 1.068, *p* = 0.30). Notably, our model showed that all males spent significantly more time in contact with their partner than the stimulus (F_(1, 34)_ = 22.745, *p* = 3.41e-5; **Supplementary Figure 1B** and **Supplementary Table 1**). Surgery treatment or interaction of main effects had no effect on our model (surgery: F_(1, 34)_ = 0.0360, *p* = 0.85; surgery x stimulus: F_(1, 34)_ = 0.0189, *p* = 0.89). *Post hoc* comparisons showed that both sham and lesioned subjects spent significantly more time in contact with their partner than with a stranger (lesioned subjects: *t*_(19.3)_ = 3.390, *p* = 0.015; sham subjects: *t*_(19.3)_ = 3.005, *p* = 0.033).


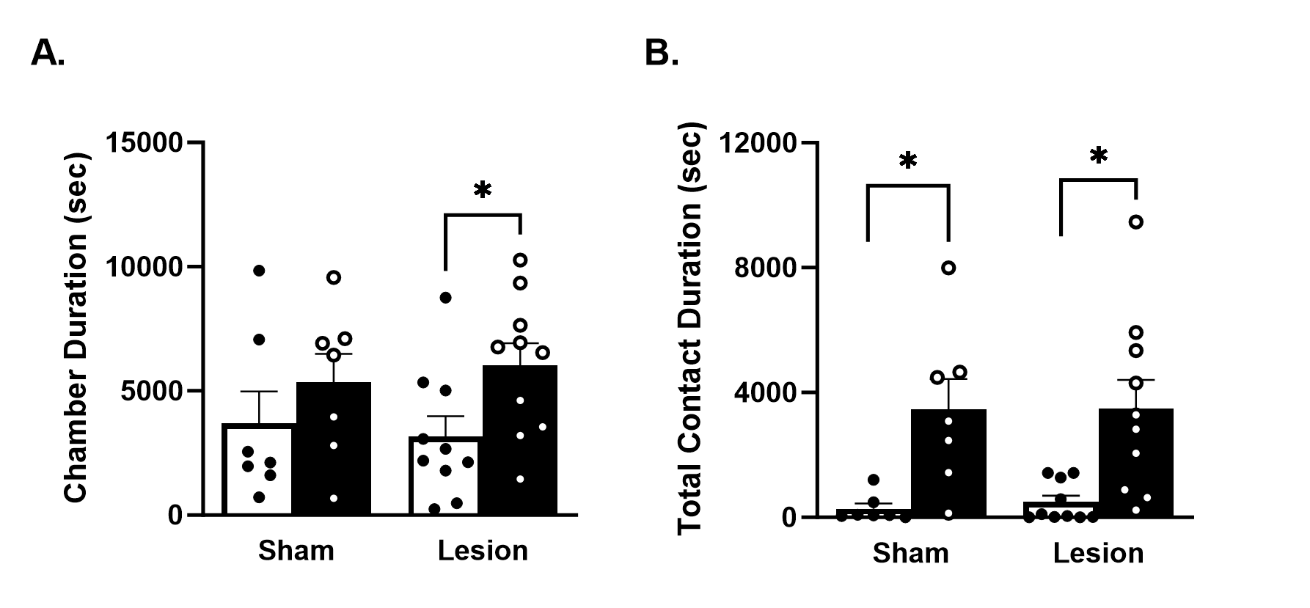


**Supplementary Figure 1 | Male pair bond formation in response to dCA1 lesions.** A) Chamber duration (sec, seconds) when sham and lesioned subjects spent time in the chamber containing either the stranger (black solid dots in white bars) or partner (black hollow dots in black bars) stimulus females. B) Total contact duration (sec) when sham and lesioned subjects spent time with the stranger (black solid dots in white bars) or partner (black hollow dots in black bars) stimulus females. Data are presented as mean ± SEM. * *p* < 0.05.
